# Antagonistic Interactions between Benzo[a]pyrene and Fullerene (C_60_) in Toxicological Response of Marine Mussels

**DOI:** 10.3390/nano9070987

**Published:** 2019-07-08

**Authors:** Audrey Barranger, Laura M. Langan, Vikram Sharma, Graham A. Rance, Yann Aminot, Nicola J. Weston, Farida Akcha, Michael N. Moore, Volker M. Arlt, Andrei N. Khlobystov, James W. Readman, Awadhesh N. Jha

**Affiliations:** 1 School of Biological and Marine Sciences, University of Plymouth, Plymouth PL4 8AA, UK; 2 School of Biomedical Sciences, University of Plymouth, Plymouth PL4 8AA, UK; 3 School of Chemistry, University of Nottingham, University Park, Nottingham NG7 2RD, UK; 4 Nanoscale and Microscale Research Centre, University of Nottingham, University Park, Nottingham NG7 2RD, UK; 5 Centre for Chemical Sciences, University of Plymouth, Plymouth PL4 8AA, UK; 6 Ifremer, Laboratory of Ecotoxicology, F-44311, CEDEX 03 Nantes, France; 7 Plymouth Marine Laboratory, Prospect Place, The Hoe, Plymouth PL1 3HD, UK; 8 European Centre for Environment & Human Health (ECEHH), University of Exeter Medical School, Knowledge Spa, Royal Cornwall Hospital, Cornwall TR1 3LJ, UK; 9 Department of Analytical, Environmental and Forensic Sciences, King’s College London, MRC-PHE Centre for Environmental & Health, London SE1 9NH, UK; 10 NIHR Health Protection Research Unit in Health Impact of Environmental Hazards at King’s College London in partnership with Public Health England and Imperial College London, London SE1 9NH, UK

**Keywords:** Trojan horse effect, B[a]P, *n*C_60_, co-exposure, mussels, DNA damage, proteomics

## Abstract

This study aimed to assess the ecotoxicological effects of the interaction of fullerene (C_60_) and benzo[a]pyrene (B[a]P) on the marine mussel, *Mytilus galloprovincialis*. The uptake of *n*C_60_, B[a]P and mixtures of *n*C_60_ and B[a]P into tissues was confirmed by Gas Chromatography–Mass Spectrometry (GC–MS), Liquid Chromatography–High Resolution Mass Spectrometry (LC–HRMS) and Inductively Coupled Plasma Mass Spectrometer (ICP–MS). Biomarkers of DNA damage as well as proteomics analysis were applied to unravel the interactive effect of B[a]P and C_60_. Antagonistic responses were observed at the genotoxic and proteomic level. Differentially expressed proteins (DEPs) were only identified in the B[a]P single exposure and the B[a]P mixture exposure groups containing 1 mg/L of C_60_, the majority of which were downregulated (~52%). No DEPs were identified at any of the concentrations of *n*C_60_ (*p* < 0.05, 1% FDR). Using DEPs identified at a threshold of (*p* < 0.05; B[a]P and B[a]P mixture with *n*C_60_), gene ontology (GO) and Kyoto encyclopedia of genes and genomes (KEGG) pathway analysis indicated that these proteins were enriched with a broad spectrum of biological processes and pathways, including those broadly associated with protein processing, cellular processes and environmental information processing. Among those significantly enriched pathways, the ribosome was consistently the top enriched term irrespective of treatment or concentration and plays an important role as the site of biological protein synthesis and translation. Our results demonstrate the complex multi-modal response to environmental stressors in *M. galloprovincialis*.

## 1. Introduction

There have been concerns regarding the potential for manufactured nanomaterials to cause unpredictable environmental health or hazard impacts, including deleterious effects across differing organismal levels, for over a decade. Despite numerous years of study, it is still unclear at what quantity manufactured nanomaterials can be found in the aquatic environment, along with their fate, potential bioavailability and subsequent hazardous effects to biological systems. This is surprising given the growing concern in the field of aquatic toxicology regarding their availability and potential toxicity [1]. Fullerenes are the smallest known stable carbon nanostructures and lie on the boundary between molecules and nanomaterials, with fullerenes generally exhibiting strong hydrophobicity in aqueous media [2]. Buckminsterfullerene (C_60_) is the only readily soluble carbon nanostructure, although graphene is dispersible in specific organic solvents [3]. Non-functionalised C_60_ possesses a measurable, but extremely low solubility in water (1.3 × 10^−11^ mg/mL), but can exist in the aqueous phase as aggregates (*n*C_60_) [4] and is quantifiable in aqueous environmental samples [5]. *n*C_60_ can be formed in water when fullerenes are released into the aquatic environment, increasing the transport and potential risk of this nanomaterials to the ecosystem ecology.

The toxicity associated with C_60_ is controversial and largely unclear [6]. The ability of C_60_ to both generate and quench reactive oxygen species (ROS) has recently been recognised as a particularly important property in the interaction of fullerenes with biological systems [7], with many aquatic studies demonstrating that fullerenes are capable of eliciting toxicity via oxidative stress [8,9,10]. Numerous studies have investigated the beneficial and toxicological effects of fullerenes [11,12,13,14,15,16,17]. However, the toxicity of nanomaterials has been shown to be dependent on numerous factors, including surface area, chemical composition and shape [18,19]. In specific cases, such as aqueous fullerenes (*n*C_60_), the physiochemical structure is influenced by different preparation methods [15,20,21]. Altered physiochemical properties induced through the different methods of solubilisation have been shown to profoundly influence the observed toxicological effects of fullerene exposure, thus making a consensus assessment of environmental toxicity difficult [20]. While the environmental toxicity of fullerenes is still being investigated, an emerging concern is whether fullerene aggregates can act as contaminant carriers (Trojan horse effects) in aquatic systems, and whether this confirms the reduction or enhancement of toxicity with these compounds. Current evidence suggests a mixture of effects dependent on chemical properties. Under combined aquatic exposure conditions (viz. *n*C_60_ and contaminant), it has been demonstrated that 17*α*-ethinylestradiol (EE2) has a decreased bioavailability [14], altered toxicity [11,22] and localised increases in mercury bioavailability [23]. Finally, when compared to other anthropogenic contaminants, Velzeboer et al. established that the absorption of polychlorinated biphenyls (PCBs) to *n*C_60_ was 3–4 orders of magnitude stronger than to organic matter and polyethylene [24]. This enhanced absorption and modifications to toxicity responses may have significant impacts on the fate, transport and bioavailability of co-contaminants already in the aquatic environment. However, more research is necessary to establish which co-contaminants bioavailability is impacted when co-exposed with *n*C_60_.

The aquatic environment is often the ultimate recipient of an increasing range of anthropogenic contaminants, and likely in all probable combinations. Organisms which are exposed to complex mixtures of differing compounds and substances can interact in many ways to induce biological responses be it additively, synergistically or antagonistically. These interactions can and do change the organismal response compared with single compound exposures [2,25,26]. Bivalves have highly developed processes for the cellular internalization of nano- and microscale particles (viz. endocytosis and phagocytosis) that are integral to key physiological functions such as cellular immunity [27]. These organisms are also useful bio-indicators because as suspension feeders they filter large volumes of water which facilitates uptake and bio-concentration of toxic chemicals [28], in addition to microalgae, bacteria, sediments, particulates and natural nanoparticles. This high filtration rate has been shown to be associated with the high potential accumulation of different chemicals in their tissues. A variety of mussel species have been used to elucidate both physiological and molecular mechanisms of action to nanoparticles [29,30] making them an ideal model to investigate how organisms respond to environmental stressors such as chemical mixtures [27]. This study aims to test the hypothesis that C_60_ fullerenes and B[a]P can interact with each other to differentially modify their potential toxicity. To confirm this hypothesis, a set of biomarkers or biological responses including proteomic analysis were employed. In this study, we hypothesized that C_60_ would act as a contaminant carrier for B[a]P and would modify the toxicity of B[a]P due to the high adsorption of B[a]P molecules onto C_60_ nano-aggregates. This hypothesis has been verified through the measurement of B[a]P and C_60_ in water and tissue. As B[a]P and C_60_ are known or potential genotoxic contaminants, a change in genotoxic effect was evaluated through the measurements of 8-oxodGuo, DNA strand breaks and DNA adducts in the digestive gland. Proteomics analysis was also performed to evaluate changes of mussels’ proteome profile under co-exposure, and to try to unravel the molecular mechanisms of the potential interactive effects.

## 2. Materials and Methods

### 2.1. Animal Collection and Husbandry

Mussels (*Mytilus galloprovincialis*; 45–50 mm) were collected from the intertidal zone at Trebar with Strand, Cornwall, UK (50° 38′ 40″ N, 4° 45′ 44″ S) in October 2016. The site has previously been used as a reference location for ecotoxocological studies and is considered relatively clean with a minimum presence of disease [31,32]. Following collection, mussels were transported to the laboratory in cool boxes and placed in an aerated tank at a ratio of 1 mussel L^−1^ with natural seawater from Plymouth Sound (filtered at 10 µm). Mussels were maintained at 15 °C, fed with micro-algae (*Isochrysis galbana*, Interpret, UK) every 2 days with a 100% water change 2 h post feeding.

### 2.2. Preparation of Stock Solutions

#### 2.2.1. Fullerenes (C_60_)

C_60_ and Er_3_N@C_80_ were obtained from Sigma Aldrich (Gillingham, UK) and Designer Carbon Materials Ltd. (Oxford, UK), respectively. In order to better replicate the conditions of the experiment during analysis, 2 mussels were maintained in 2 L glass beakers for 24 h with natural seawater from Plymouth Sound (filtered at 10 μm). Subsequently, fullerenes (1 mg) were added to the mussel-exposed seawater (10 mL) and the suspension homogenised by ultrasonication (Langford Sonomatic 375, Bromsgrove, UK, 40 kHz) for 1 h at room temperature. The suspension was allowed to settle for at least 4 h at room temperature prior to analysis of the aggregate size. Dynamic light scattering (DLS) was performed using a Malvern Zetasizer Nano-ZS (Malvern, UK) at room temperature. Quoted values are the average of 3 measurements. Bright field transmission electron microscopy (TEM) and dark-field scanning transmission electron microscopy (STEM) were performed using the JOEL 2100+ microscope (Welwyn Garden City, UK) operated at 200 keV. Energy dispersive X-ray (EDX) spectra were acquired using an Oxford Instruments INCA X-ray microanalysis system (Oxford, UK) and processed using Aztec software (version 3.1 SP1, Oxford, UK). Samples were prepared by casting several drops of the respective suspensions onto copper grid-mounted lacey carbon films.

#### 2.2.2. Benzo[a]pyrene (B[a]P)

B[a]P (≥96%, B1760, Sigma Aldrich) is not water soluble and was previously dissolved in dimethyl sulfoxide (DMSO) after having determined its solubility limit. Chemical solutions were prepared so that the DMSO concentration in the sea water was 0.001%.

### 2.3. In Vivo Exposure of M. galloprovincialis to B[a]P and C_60_: Experimental Design

Following depuration, mussels were separated (2 per beaker) into 2 L glass beakers containing 1.8 L of seawater and allowed to acclimatize for 48 h. A photoperiod of 12 h light: 12 h dark was maintained throughout the experiment. Oxygenation was provided by a bubbling system. Seawater was monitored in each of the beakers by measuring salinity (36.45 ± 0.19‰). Mussels were exposed for 3 days with no water changes to B[a]P (5, 50 and 100 µg/L), C_60_ alone (0.01, 0.1 and 1 mg/L) and a combination of B[a]P (5, 50 and 100 µg/L) and C_60_ (1 mg/L). Control groups received only DMSO at the same concentrations as used in the other exposure groups (0.001% DMSO). A total of 26 individuals were used per treatment. Following exposure, tissue samples were collected as follows: gill and digestive gland (DG) tissue was collected from 3 mussels for chemical analysis, digestive tissue was collected from 9 mussels and pooled (3 mussels per one biological replicate) for shotgun proteomics, DG tissue from 10 mussels was collected for comet assay and DNA adducts, with a further 5 DG collected for DNA oxidation. Water samples from 3 beakers were randomly collected during each treatment for B[a]P and C_60_ analyses.

### 2.4. Gas Chromatography–Mass Spectrometry (GC–MS) Analyses of B[a]P in Water and Tissue

Water and tissue extracts were analysed using an Agilent Technologies (Stockport, UK) 7890A Gas Chromatography (GC) system interfaced with an Agilent 5975 series Mass Selective (MS) detector as described in [33].

### 2.5. Analyses of C_60_ in Water and Tissue

The analyses of C_60_ were performed on the toluene extracts common to the B[a]P analyses. The water extracts were analysed with an Agilent 1100 high-performance liquid chromatography-ultraviolet-visible instrument (HPLC-UV, Stockport, UK). The separation was performed on a Shimadzu XR-ODS column (particle size 2.2 µm, 3.0 × 50 mm, Milton Keynes, UK) using an acetonitrile-toluene gradient starting at 40% toluene, at a flow rate of 1 mL/min and a column temperature set at 40 °C. The detection wavelength was set at 330 nm and the fullerene absorption at maximum. Quantification was performed by external calibration using authentic fullerene standards. Because of their lower concentrations, the tissue extracts were analysed by ultrahigh performance liquid chromatography coupled with high resolution mass spectrometry following a protocol adapted from [34].

### 2.6. Proteomics

#### 2.6.1. Sample Collection and Quality Check

Tissue was removed from the −80 °C, weighed (100 mg) and twice washed in phosphate buffered saline (PBS) prior to being homogenised on ice for 60 s in radioimmunoprecipitation assay (RIPA) buffer. The lysed homogenate was centrifuged at 14,000 RPM for 60 min at 4 °C, the supernatant collected and aliquoted. Protein concentration was determined using the Pierce BCA protein assay kit (Thermo Scientific, Loughborough, UK) according to manufacturers’ instructions with bovine serum albumin as standard. Reproducibility of protein extraction was carried out using sodium dodecyl sulfate polyacrylamide gel electrophoresis (SDS-PAGE). Briefly, 30 µg of protein from each sample was loaded on a polyacrylamide gradient gel (4–12%) and stained with Coomassie protein stain (Expedeon, UK) and destained with ELGA water. Quality checked protein samples were then processed for downstream liquid chromatography-mass spectrometry (LC-MS, Stockport, UK) analysis.

#### 2.6.2. Sample Preparation for LC-MS

Equal amounts of intestinal protein (100 µg) were processed using the Filter Aided Sample Preparation (FASP) method as described by [35]. The digested proteins were subsequently purified using the Stop-and-go-extraction (STAGE) tip procedure as previously described [36]. Tryptic peptides were analysed using LC-MS.

#### 2.6.3. Mass Spectrometry

Peptides were separated on a Dionex Ultimate 3000 RSLC nano flow system (Dionex, Camberly, UK) and analysed as described in [37].

#### 2.6.4. Analysis

*Peptide identification and quantification.* Data analysis and quantification was performed using R (Version 3.5.0, Vienna, Austria) [38]. Thermo .raw files were imported into ProteoWizard [39] and converted to .mzML format before identification using the MS-GF+ algorithm which is implemented in R via the MSGFplus package [40]. MS-GF+ was chosen due to its known sensitivity in identifying more peptides than most other database search tools and its ability to work well with diverse types of spectra, configurations of instruments and experimetnal protcols [41]. The protein database utilised in this study consisted of the UniProt KnowledgeBase (KB) sequences from all organisms from the taxa Mollusca, sub category Bivalvia (84,410 sequences released 1/10/2018). This was cocatenated with a common contaminants list downloaded from ftp://ftp.thegpm.org/f asta/cRAP (Version: January 30th, 2015) using the R package seqRFLP [42]. Searches were carried out using the following criteria: mass tolerance of 10 ppm, trypsin as the proteolytic enzyme, maximum number of clevage sites = 2 and cysteine carbamidomethylation and oxidation as a fixed modification. Target decoy approach (TDA) was applied as it is the dominant strategy for false discovery rate (FDR) estimation in mass-spectrometry-based proteomics [43]. A 0.1% peptide FDR threshold was applied in accordance with standard practice, with a 1% protein FDR applied after protein identification (via aggregation). The resulting .mzid files were converted to MSnSet and quantified using label free spectral counts. The mass spectrometry proteomics data have been deposited to the ProteomeXchange Consortium [44] via the PRIDE [45] partner repository with the dataset identifier PXD013805 and 10.6019/PXD013805.

*Data processing and quantification*. Data processing was undertaken as follows: each sample was run individually and then regionally combined before all samples were amalgamated into a large dataset. Quantification of proteins occurred via spectral index (SI) [46]. For identification of proteins, the common practice of requiring three peptides per protein was used in order to reduce the number of false positives [47]. Peptides were subsequently aggregated using sum and the protein intensities scaled based on the actual number of proteins summed. Mussel samples were grouped based on biological replicate, exposure and concentration and the resulting data filtered to keep proteins which were identified in more than two biological replicates. To quantitatively describe reliable and biologically relevant protein expression changes based on single exposure to B[a]P, C_60_ or to a combination of the two, the data analysis was split into three distinct sections. As per recent recommendations, normalisation was carried out first [48]. Based on systematic evaluations of normalisation methods in label free proteomics, normalisation between technical replicates was carried out using variance stabilization normalisation (Vsn) [49]. Based on a study by Lazar et al. [48], it was hypothesized the most likely cause of missing values will be due to a mixture of MAR (missing at random), MCAR (missing completely at random) and MNAR (missing not at random) data. As such, missing value imputation was carried out using a mixed methodology in the form of KNN (K nearest neighbours, biological replicates) and QRILC (left censor method for MNAR data; whole dataset) [50,51]. Following normalisation, differential expression was carried out using msmsTests [52] with *p*-value less than 0.05 considered significant and Q-values (FDR: <1%) calculated for *p*-value target matches with the Benjamini–Hochberg procedure. Enrichment of function among up- or downregulated proteins was calculated using GOfuncR using gene ontologies associated with differentially expressed proteins (P-adj = 0.01, calculated using Benjamini–Hochberg method and q-value = 0.05). Kyoto Encyclopaedia of Genes and Genomes (KEGG) analysis was carried out on the identified unique proteins per treatment (*p* < 0.05) using the clusterProfiler package [53]. KEGG annotation was performed using GhostKOALA [54] and pathways with significant enrichment identified using ClusterProfiler (hypergeometric test, *q* < 0.05 following Benjamini correction). Unique and common proteins based on toxicant were graphically represented through Venn diagrams with the software Venny (http://bioinfogp.cnb.csic.es/tools/venny/index.html) [55]. The R script outlining project analysis for this study can be found in supplementary materials (R script S1).

### 2.7. DNA Damage

#### 2.7.1. Measurement of 8-oxodGuo Levels Using HPLC/UV-ECD

DNA extraction was performed using 20 mg of digestive gland tissue according to the chaotropic NaI method derived from Helbock et al. [56], slightly modified by Akcha et al. [57]. In addition, 8-oxodGuo levels were determined by HPLC (Agilent 1200 series, Les Ulis, France) coupled to electrochemical (Coulochem III, ESA, Illkirch, France) and UV (Agilent 1200 series) detection as described in [58].

#### 2.7.2. Comet Assay

The comet assay on digestive gland tissue was performed as previously described in [33].

#### 2.7.3. DNA Adducts

For each sample, DNA from gills and DG tissues was isolated using a standard phenol-chloroform extraction procedure. We used the nuclease P1 enrichment version of the thin-layer chromatography (TLC) ^32^P-postlabelling assay [59] to detect B[a]P-derived DNA adducts (i.e., 10-(deoxyguanosin-*N*^2^-yl)7,8,9-trihydroxy-7,8,9,10-tetrahydro-B[a]P [dG-*N*^2^-BPDE]). The procedure was essentially preformed as described [59]. After chromatography, TLC sheets were scanned using a Packard Instant Imager (Dowers Grove, IL, USA) and DNA adduct levels (RAL, relative adduct labelling) were calculated as reported [60]. An external BPDE-modified DNA standard was used as a positive control [61].

### 2.8. Confirmation of Uptake of Fullerenes by Mussels

#### 2.8.1. Experimental Design

Mussels were exposed to a single treatment, 1 mg/L Er_3_N@C_80_ for 3 days (static exposure). For each treatment (control and labelled fullerenes), 2 mussels were exposed into 2 L glass beakers containing 1.8 L of seawater.

#### 2.8.2. Bulk Spectroscopic Analysis

For the determination of erbium concentration in the digestive gland, 2 mussels per treatment were analysed using an X Series II ICP-MS (Thermo Fisher Scientific Inc., Waltham, MA, USA) with PlasmaLab software (Thermo Fisher Scientific Inc., Waltham, MA, USA) as described in [32].

#### 2.8.3. Mussel Sectioning and Electron Microscopy Analysis

Following the exposures detailed above, a small piece (~5 mm^2^) was dissected out of the centre of the digestive gland and fixed in 2% paraformaldehyde, 2.5% glutaraldehyde, 2.5% NaCl, 2 mM CaCl_2_ in 0.1 M PIPES, pH 7.2 for 3 h. The tissue was then stored in 2.3 M sucrose (in 0.1M PIPES) until analysis. Two mussels were analysed per treatment. Electron transparent sections for scanning transmission electron microscope (STEM) analysis were prepared by cutting ~1 mm^2^ pieces from the washed whole tissues and sectioning to a thickness of ~180–200 nm at −80 °C using the RMC Products PowerTome with the CR-X cryochamber (Tucson, AZ, USA). The cross-sections were transferred onto copper-grid mounted graphene oxide films using the Tokuyasu technique and imaged in dark field STEM using the JOEL 2100+ microscope operating at 200 keV.

### 2.9. Statistical Analysis

Statistical tests were conducted using R software (Version 3.6.0, Vienna, Austria) [62]. Normality and variance homogeneity were evaluated using Lilliefor’s test and Bartlett’s test, respectively. When necessary, raw data were mathematically transformed (Ln) to achieve normality before proceeding with an ANOVA. When significant, a posteriori Tukey test was performed. When data could not be normalized, statistical differences between treatments were tested using the non-parametric Kruskal–Wallis test.

#### Analysis of Interactions

Further analysis of the combined effects of C_60_ and BaP on DNA Damage (based on Comet Assay) was performed by calculating the Interaction Factor (IF) in order to test for evidence of additivity, synergism and antagonism [63,64,65]:

IF = (G_(C60 + BaP)_ − C) − [(G_(C60)_ − C) + (G_(BaP)_ − C)] = G_(C60 + BaP)_ − G_(C60)_ − G_(BaP)_ + C, (1)

SEM_(IF)_ = √(SEM^2^_(C60 + BaP)_ + SEM^2^_(C60)_ + SEM^2^_(BaP)_ + SEM^2^_(C)_), (2)

where IF is the interaction factor: negative IF denotes antagonism, positive IF denotes synergism, and zero IF denotes additivity. G is the mean cell pathological reaction to toxicants (BaP, C_60_ and BaP + C_60_), and C is the mean cellular response under control conditions. SEM_(x)_ is the standard error of the mean for group X. Results were expressed as IF, and the 95% confidence limits were derived from the SEM values.

In order to test the mixture IF values against predicted additive values (assumed to have an IF = 0), the predicted additive mean values (A) were calculated:

A = (G_(C60)_ − C) + (G_(BaP)_ − C). (3)

The Pythagorean theorem method for combining standard errors was used to derive combined standard errors for the predicted mean additive values (A) of C_60_ and BaP (http://mathbench.org.au/statistical-tests/testing-differences-with-the-t-test/6-combining-sds-for-fun-and-profit/). The standard errors for the three C60 and BaP treatments (predicted additive) were derived using the following equation:

SEM_(add)_ = √(SEM^2^_(C60)_ + SEM^2^_(BaP)_ + SEM^2^_(C)_). (4)

This enabled the 95% confidence limits to be derived for the predicted additive values. The confidence limits were used to test the predicted additive values having an IF = 0 against the IF values for the mixtures.

## 3. Results and Discussion

Bivalves are ideal organisms for evaluating the adverse effects caused by various environmental stressors including polycyclic aromatic hydrocarbons (PAHs) and nanomaterials. PAHs such as B[a]P have a ubiquitous aquatic distribution and are known to cause several adverse effects in a diverse range of aquatic organisms. Nanomaterials, both as solids and colloids, are ingested by many organisms and bio-accumulate in large quantities, especially in molluscs. The mussel digestive gland is one of the principal detoxification organs with an acknowledged concentration of phase I detoxification enzymes [66]. As such, it is unsurprising that a mussel digestive gland has been used as model tissue for eco-toxicological studies of various NPs [67,68,69], with Di et al. reporting that the digestive gland in *Mytilus edulis* accumulates more C_60_ than other tissues [67].

There is considerable debate in the literature regarding the actual toxicological impact of nanomaterials in the aquatic environment, with fullerene toxicity controversial. In the aquatic system, Kahru et al. compiled fullerene toxicological data for fourteen organisms and classified C_60_ as “very toxic” [70]. Using mouse and human cell lines, Isakovic et al. demonstrated that pristine C_60_ and aqueous suspensions of C_60_ are more toxic than its hydroxylated derivatives [71]. In marked contrast, other studies have demonstrated that pristine C_60_ has low or limited toxicity to cells and various organisms [10,72,73,74]. The lack of consensus regarding C_60_ toxicity may be partly due to limited studies which incorporate both a physiological and ecological approach. As a consequence, little is still known about NP bioavailability, mode of uptake, ingestion rates and actual internal concentrations related to Absorption, Distribution, Metabolism and Excretion (ADME) [27]. Despite the contradictory reports, there is consensus that some nanomaterials may potentially affect biological systems directly but also through interactions with other compounds that may be available in the environment (reviewed in [6]). Studies that investigate co-exposure with carbon-based nano-compounds, such as nanotubes or fullerenes are limited, especially in aquatic systems. Using *Danio rerio* (zebrafish) hepatocytes, Ferreira et al. investigated the co-exposure of C_60_ with B[a]P and provided evidence of toxicological interactions, whereby C_60_ increased the uptake of B[a]P into cells, decreased cell viability and impaired detoxification responses [75], while Baun et al. reported that co-exposure with fullerene C_60_ enhanced toxicity of phenanthrene to *Daphnia magna* and *Pseudokirchneriella subcapitata* [22]. With respect to B[a]P and C_60_, Di et al. demonstrated organ specific response to both single and combined mixtures with no observation of cytoxicity and duration dependent and condition specific genotoxic response in *M. galloprovincialis* [67]. Importantly, the observed genotoxic response was reversible after a recovery period. While single exposure studies are more common, bivalve species have already been used as biological models in proteomics to assess the effects of complex mixtures [22,76,77]. However, proteomics analyses on combined exposure with carbon nanomaterials in aquatic organisms are still very scarce [9].

### 3.1. Characterization of C_60_ in Seawater

Dynamic light scattering and electron microscopy analysis (Figures S1–S3 and Table S1) of C_60_ dispersed in mussel-exposed seawater (~100 μg/mL) with brief ultrasonication followed by equilibration indicates the formation of stable aggregates measuring 653 ± 87 nm (*n*C_60_ where *n* = ~2 × 10^8^) in mean hydrodynamic diameter. No significant change in the size of *n*C_60_ aggregates was observed upon addition of B[a]P.

### 3.2. Assessement of the Interaction between C_60_ and B[a]P through Bioaccumulation in Gills and the Digestive Gland

Changes in the bioavailability of contaminants co-exposed with carbon nanomaterial have been reported, from a decrease in bioavailability [78,79] to its enhancement, also called the “carrier effect” [80,81]. It has been demonstrated that carbon nanopowder helps B[a]P uptake by zebrafish embryos and very interestingly also affected the distribution of the pollutant in the organism [82]. However, in the same species, in zebrafish larvae, it has been shown that bioavailability of 17α-ethynylestradiol (EE2) was reduced with increasing concentration of *n*C_60_ nanoparticles [14].

**Figure 1:**
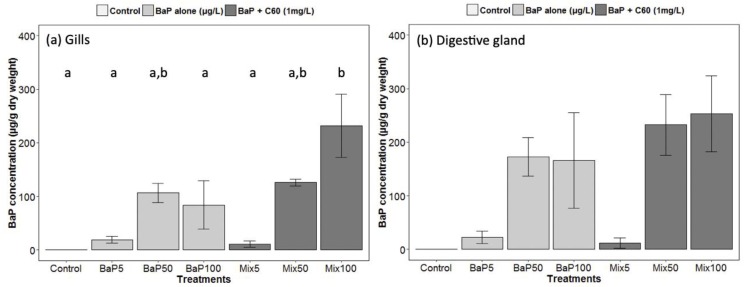
Gas Chromatography−Mass Spectrometry (GC–MS) analyses of B[a]P in (**a**) gills and (**b**) digestive gland of *M. galloprovincialis*. Data marked with different letters differed significantly (Tukey post-hoc test; *p* < 0.05).

In our study, we analyzed for the first time in a marine bivalve, B[a]P uptake (at different exposure concentrations) in the digestive gland in the presence of C_60_ fullerene in order to highlight a possible role of contaminant carrier of C_60_. Regarding analyses of B[a]P in seawater, nominal concentrations were matched to stock concentrations (Table S2). No difference was observed between the presence or absence of C_60_. As already established [33], there was a rapid disappearance of B[a]P over time in seawater and B[a]P accumulated preferentially in the digestive gland tissue. Interestingly, comparable B[a]P tissue concentrations in the presence or absence of C_60_ were observed indicating that, despite the expected strong sorption of B[a]P on C_60_ [83], no Trojan horse effect was observed and C_60_-sorbed B[a]P remains also bioavailable to *M. galloprovincialis* (Figure 1). In gills, a significantly higher uptake is observed in the presence of C_60_ at the highest concentration of B[a]P (Figure 1). In general, high variability would conceal subtle changes. It appears that the bioavailability of nanomaterials and their co-contaminants depend on many factors such as their size, shape, surface coating and aggregation state and on the metabolism of the species investigated [78,84].

**Figure 2:**
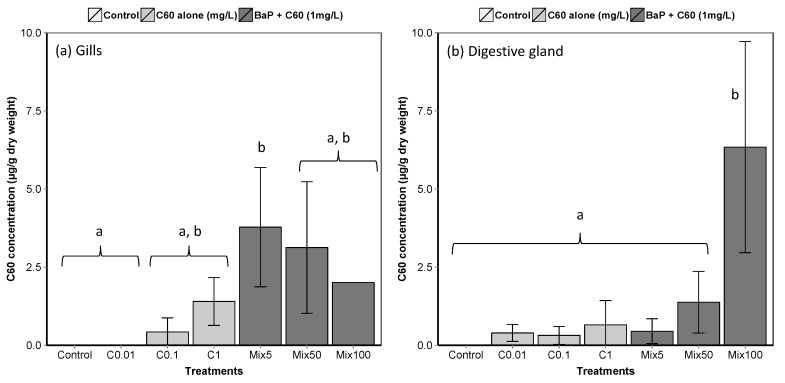
Liquid chromatography-mass spectrometry (LC–MS) analyses of C_60_ in *M. galloprovincialis* (**a**) gills and (**b**) digestive gland (means ± SE). Data marked with different letters differed significantly (Tukey post-hoc test; *p* < 0.05). An analytical problem led to the loss of two samples of the gills from mussels exposed to Mix100 explaining the absence of standard error.

A rapid decline in the concentration of C_60_ in seawater was observed with time, with no quantifiable amounts after day 1 (Table S3). At t_0_, the measured water concentrations are in reasonable agreement with the nominal concentrations (427.6 ± 45.3, 63.8 ± 11.9 and 7.3 ± 1.8 µg/L for nominal concentrations of 1000, 100 and 10 µg/L, respectively). Low but quantifiable amounts of C_60_ in *M. galloprovincialis* tissues indicate active uptake, with adsorption on the outside of the tissue ruled out due to external washes with toluene prior to analysis (Figure 2). High variability in C_60_ concentrations in gills and DG makes it difficult to detect a difference in accumulation between treatments and to conclude regarding the uptake of C_60_ by mussels.

To provide further insight into the uptake of fullerenes by marine mussels, it was necessary to use a form labelled with a diagnostic marker. In our experiments, we explored the application of the endohedral fullerene Er_3_N@C_80_, fabricated using the trimetallic nitride template (TNT) process, as it represents a good structural analogue to C_60_, possessing similar surface chemistry, and contains a rare earth element, shielded from the external environment within the fullerene cage, which is not found in nature. The presence of erbium in the mussel digestive gland, as a diagnostic of the uptake of labelled fullerenes, was thus quantified using Inductively Coupled Plasma Mass Spectrometer (ICP–MS) and found with a mean concentration of 151.5 μg/kg (236.5 and 66.4 μg/kg for each mussel). However, despite an exhaustive electron microscopy investigation of whole and cross-sectioned DG tissues (Figure S4, Supplementary materials), no direct visualisation of labelled fullerenes was observed. This result indicates that the fullerenes are likely distributed within the tissues at the near molecular level (i.e., highly dispersed) and therefore below the sensitivity of either microscopy or in situ spectroscopy approaches in complex materials such as these. In a previous study in *M. galloprovincialis* [85], it has been showed that mussels exposed to C_60_ alone exhibited higher accumulation of C_60_ in the digestive gland compared to the gill. Interestingly, co-exposure to fluoranthene modified accumulation of C_60_, with higher accumulation of C_60_ when animals are exposed to C_60_ alone compared to combined exposure.

When comparing water and tissue concentrations for B[a]P and C_60_, the bioconcentration observed in our conditions was much lower for C_60_ compared to B[a]P: the uptake in the DG of mussels exposed to a similar aqueous concentration of B[a]P and C_60_ was about 2000 times more important for B[a]P. However, non-constant concentrations in the aqueous phase, attributed to sorption and/or sedimentation, did not allow the calculation of bioaccumulation factors, which also requires reaching a steady-state in the tissues. The difference between B[a]P and C_60_ tissue concentration could also be attributed to different kinetics of uptake, which could only be explored through longer exposure periods and regular sampling. Recent work indicated a continuous increase of C_60_ concentrations in whole mussels over at least three weeks [86].

### 3.3. Assessment of the Interactive Effect of C_60_ and B[a]P through Genotoxicity

**Figure 3:**
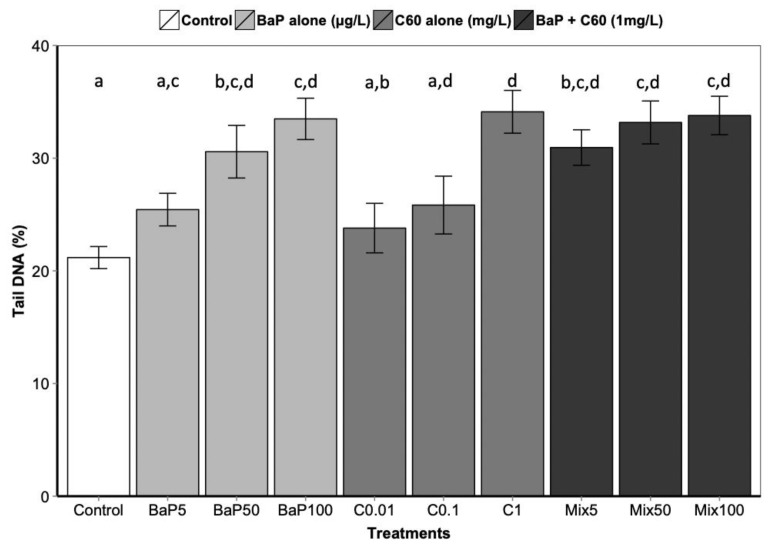
DNA strand break level following 3 days of exposure to C_60_, B[a]P and mixture of both in the digestive gland. Data marked with different letters differed significantly (Tukey post-hoc test; *p* < 0.05).

**Figure 4:**
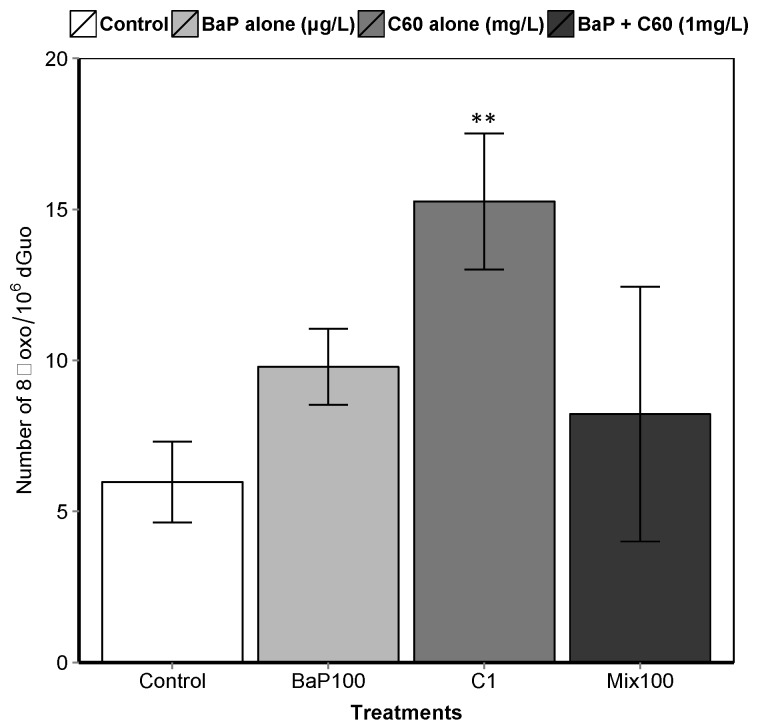
8-oxodGuo levels in the digestive gland of mussels. Asterisks indicate the statistical differences observed between control and exposed groups. (**) *p* < 0.01.

B[a]P is a known genotoxic, mutagenic and carcinogenic [87]. According to a review by Johnston et al., fullerene toxicity has been suggested to involve oxidant-driven response and suggests evaluating toxicity by including oxidative stress and related consequences including inflammation or genotoxicity [88]. We assessed the interactive effect of C_60_ and B[a]P through three different genotoxicity assays. Regarding single exposures, B[a]P induced DNA strand breaks in the digestive gland at the intermediate and highest concentrations (50 and 100 μg/L) after three days of exposure (Figure 3). No effect on DNA strand breaks was observed at the lowest concentration. No effect was also observed on the level of 8-oxodGuo for B[a]P treatment. These results could be due to the short exposure time (three days). In [57], an increase in the level of 8-oxodGuo was observed after 10 days of B[a]P exposure in the digestive gland of *M. galloprovincialis*. Regarding exposure to C_60_ only, higher DNA strand breaks compared to the controls were observed only at the highest concentration (1 mg/L, *p* < 0.001). A significant increase (*p* = 0.00108) in 8-oxo-dGuo levels was also detected in the digestive gland of mussels exposed to C_60_ (15.3 ± 2.3) compared to control (5.9 ± 1.3) (Figure 4). Lower C_60_ concentration did not appear to have any genotoxic effects (DNA strand breaks) on mussel digestive gland at the concentrations tested. Whatever the exposure concentration of B[a]P and mixture of B[a]P and C_60_, no DNA adducts were detectable in DNA samples from the digestive gland of *M. galloprovincialis*.

As observed for the bioaccumulation of contaminants co-exposed with carbon nanomaterial, controversial results are also obtained in the literature regarding genotoxicity. In aquatic organisms, co-exposure to C_60_ and organic contaminants induced a range of responses, to no effect until synergistic and antagonistic responses compared to single exposure [9,67,85]. In our study, no significant differences in DNA strand breaks were observed between exposure to B[a]P or C_60_ alone compared to co-exposure (Figure 3). Interestingly, the analysis of interactions performed on the comet assay and the oxidative DNA damage results revealed an antagonistic interaction only at the highest concentration between C_60_ and B[a]P (Table 1). This antagonistic effect may be caused by a reduction in ROS generation, or more effective scavenging of ROS by C_60_, when C_60_ and B[a]P are present together in close association, as previously described by [9,67]. C_60_ fullerenes are both scavengers and generators of reactive oxygen species (ROS) [89]; and when C_60_ and B[a]P are closely associated or bound together within the lysosomal compartment of the mussel digestive cells, their ROS scavenging and generating properties may be altered.

### 3.4. Assessment of the Interactive Effect of C_60_ and B[a]P on the Proteome Profile of the Digestive Gland

Investigations into proteome responses of marine organisms to various stressors is comparatively small when compared to other model laboratory organisms, both aquatic and terrestrial. Proteomic analysis represents a fundamental step in extending understanding of the physiological processes involved in organismal responses to environmental stressors. In addition, proteomics also provides better qualitative data on post-translational modifications without interference from mRNA instability [90]. A major limitation in the field has been the lack of available annotated genomes for a broad diversity of marine organisms. As a consequence, it has been considered a widely under utilised tool [91]. The lack of genome information has not stopped studies on proteome characterisation in bivalvia/mollusca species using broad protein databases limited to either the phylum, class or specific combination of species [92,93,94,95]. However, studies investigating proteome response to environmental stressors or injury are less abundant [30,77,96]. In the current study, a label free shot-gun proteomics approach was performed for the first time to our knowledge in aquatic organisms to investigate proteome alterations following treatment with B[a]P and C_60_ alone and a combination of B[a]P with 1 mg/L of C_60_. This untargeted method was specifically chosen to identify molecular pathways involved in the interaction of C_60_ and B[a]P without a priori assumptions.

#### 3.4.1. Identification of Differentially Expressed Proteins

In order to identify differentially expressed proteins in the digestive gland proteome of controls, B[a]P, nC_60_ and mixture (B[a]P and 1 mg/L nC_60_), a label free LC-MS/MS approach was used with trypsinised tissue homogenates. Following removal of common contaminants in each dataset, peptide mapping quantified 3125, 3428 and 3475 unique proteins following identification from the Universal Protein Resource (UNIPROT) database distinct to B[a]P, C_60_ and mixture (B[a]P and 1 mg/L C_60_) treatments, respectively. Irrespective of treatment, protein sequences from the Pacific oyster *Crassostrea gigas* (Organism ID = 94323) were highly represented in the samples at approximately 38%, followed by Japanese scallop *Mizuhopecten yessoensis* (Organism ID = 6573) at 34%. Surprisingly, sequences from the genus *Mytilus* were less represented in the search at approximately 3% with the Mediterranean mussel *Mytilus galloprovincialis* (Organism ID = 29158) representing approximately 1% of identified sequences. This may be due to a lack of genomic information available for this genus in the UNIPROT database, even though a genome sequence is available [97].

**Figure 5:**
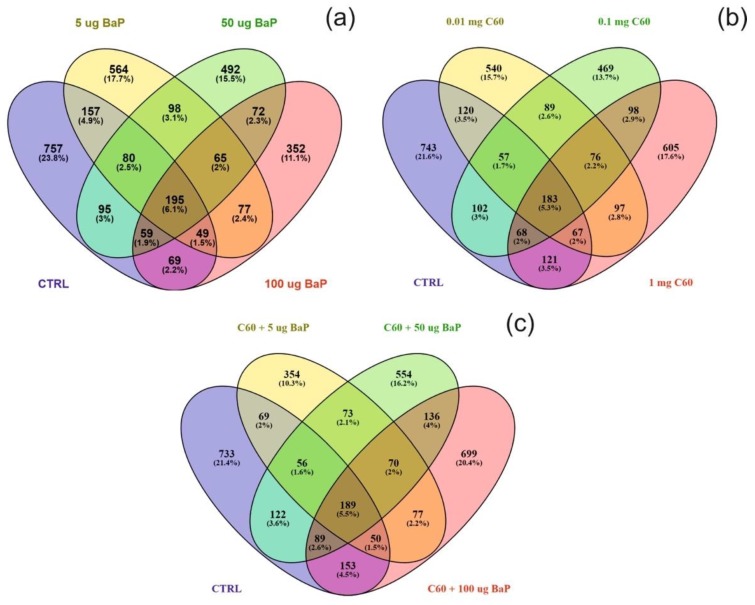
Venn diagram visualising the overlap between the control sample and varying concentrations of B[a]P (**a**), C_60_ (**b**) or a mixture of the two (5–50–100 µg/L B[a]P 1 mg/L C_60_) (**c**) following exposure for three days. Note that overlap is based on a threshold of *p <* 0.05 and does not include FDR correction.

**Figure 6:**
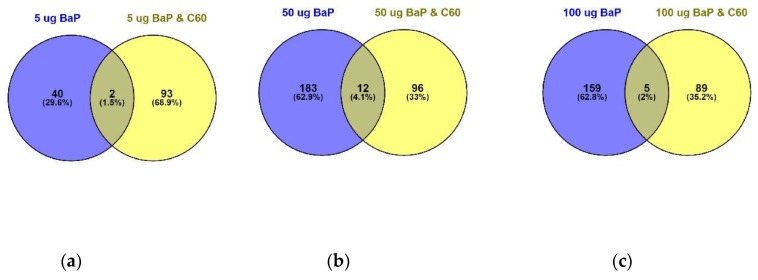
Venn diagram visualising the overlap between 5 µg/L (**a**), 50 µg/L (**b**) and 100 µg/L (**c**) of B[a]P with a mixture solution containing the same B[a]P concentrations in addition to 1 mg/L of C_60_ following 24 h exposure. Overlap is based on *p* < 0.05 and FDR set at 1%.

Differentially expressed proteins (DEPs) were determined using a quasi-likelihood GLM. Comparison of each dose per treatment (B[a]P: 5, 50 and 100 µg/L, nC_60_: 0.01, 0.1 and 1 mg/L, and a mixture: 5, 50 and 100 µg/L B[a]P and 1 mg/L *n*C_60_) with the control group was visualised using Venn diagrams (Figure 5). Minimal overlap between varying concentrations was observed for the mixture treatment (average of 2%) (Figure 5c) when compared to B[a]P (Figure 5a, 9%) or nC_60_ (Figure 5b, 8%). Volcano plots were used to visualise statistically significant changes in protein abundance for varying concentrations of the above treatments following comparison to controls (Figure S5). Applying a 1% FDR threshold, 401 differentially expressed proteins were identified following B[a]P treatment (all concentrations) and 297 differentially expressed proteins were identified following treatment with the mixture of B[a]P and nC_60_. No differentially expressed proteins (*p* < 0.05) were identified in C_60_ treated samples. The identified DEPs can be further broken down based on treatment with 42, 50 and 164 DEPs identified at 5, 50 and 100 µg/L B[a]P. Following exposure to a mixture solution, 95, 108 and 94 DEPs were identified at each concentration respectively (1 mg/L of C_60_ and 5, 50 and 100 µg/L of B[a]P) with Figure 6 representing a visual comparison of commonalities between single exposure versus combined exposure. A subset of DEP based on the top three unique proteins per concentration is displayed in Table 2, with the full list of unique proteins and associated *p*-value and FDR correction (Spreadsheet S1). The majority of differentially expressed proteins detected in this study (B[a]P and mixture exposure) were downregulated (52%) between the treatment and control conditions irrespective of concentration.

The trend towards higher protein alterations in single exposures versus co-exposures suggests a non-additive combine effect and is in agreement with prior studies which reported generally higher protein alterations of B[a]P and Cu under single exposure then when co-exposed together [77]. The data in this study suggest that an interaction occurs between B[a]P and C_60_ whereby the effect of the mixture is different from the presumption of additivity (were by dose response relationships of mixtures are enhanced in comparison to the individual components) as outlined in Rosa et al. [98]. In this case, the data suggests an antagonistic relationship between B[a]P and C_60_ at the higher concentrations of 50 and 100 µg/L. This observation has previously been observed in *Mytilus edilus* digestive gland [67]. However, this trend is not replicated at the lowest concentration of 5 µg/L whereby mixture exposure resulted in higher DEPs than single exposure. This difference in DEPs may potentially be related to reduced accumulation of B[a]P at the higher concentrations due to saturation of mussel tissue and thereby limiting protein changes. In previous studies, increased impact and accumulation of B[a]P at lower concentrations in *M. galloprovincialis* have been attributed to tissue saturation [99]. The increase in differentially expressed proteins at the lower concentration may also reflect the inability of membrane transporters such as p-glycoprotein to efflux this particular nanoparticle [100] and as such acts to bypass typical protective mechanisms initiated to protect the organism from PAH stress.

#### 3.4.2. GO Functional Enrichment

Gene ontologies were directly annotated using a custom annotation database derived from UNIPROTKB (bivalvia) with enrichment carried out using GOfuncR. This provides a controlled vocabulary to describe gene product characteristics in three independent ontologies viz. biological process, molecular function and cellular components. Based on the R package GOfuncR, 31, 35 and 23 GO nodes were found enriched at a threshold of *p <* 0.05 (Family wise error rate (FWER) correction) following treatment with B[a]P, C_60_ or co-mixtures (5–100 µg/L B[a]P and 1 mg/L C_60_). The top GO terms are listed in Table 3 (threshold set FWER = 0.01), while the full list separated by treatment and concentration can be found in supplemental material (Spreadsheet S1). Irrespective of treatment, biological process records the majority of enriched terms. The ability of a stress organism to adjust its cellular processes via transcriptional and subsequently proteomic processes allows it where possible to minimise cellular damage, which may lead to organism death. GO analysis revealed 30 enriched proteins following B[a]P exposure, 42 following C_60_ exposure and 31 in the mixture exposure. The response of *M. galloprovincialis* to B[a]P is characterised by a predominant enrichment of Biological processes (67% or 20 GO’s) with the majority of these occurring at 100 µg/L. When compared to the mixture model at the same concentration, seven terms are absent in the mixture model compared to the single exposure viz. DNA metabolic processes (GO:0006259), DNA repair (GO:0006281), Cellular response to DNA damage stimulus (GO:0006974), cellular response to stress (GO:0033554), metabolic processes (GO:0008152), cellular metabolic processes (GO:0044237) primary metabolic processes (GO:0044238) and organic substance metabolic processes (GO:0071704). The absence of these enriched terms at the highest mixture concentration of B[a]P and C_60_ in association with the reduction in differentially expressed proteins (when compared to single exposure and 50 µg/L) suggest an antagonistic interaction between the two common contaminants. This may be explained by known properties of the chemicals. *n*C_60_ is an exceptional free radical scavenger [101,102], while B[a]P has been shown to produce free radicals under a variety of conditions [103]. B[a]P contributes approximately 50% of the total carcinogenic potential of the PAH group [104]. Transcriptomic alterations related to B[a]P are likely to be related to genotoxic mechanisms in addition to other biological processes such as mitochondrial activities and immune response as outlined previously [33]. In contrast, Zhang et al. demonstrated that aqueous C_60_ aggregates induced apoptosis of macrophage by changing the mitochondrial membrane potential [105]. As predicted by the literature, enriched GO terms following single *n*C_60_ exposure are predominantly related to changes to the membrane-enclosed, organelle and intracellular lumen, while mixed exposure resulted in enrichment of mitochondrial components (viz. matrix, ribosome and protein complex). This enrichment of organelle cellular components correlates with enrichment of the ribosome KEGG pathway (ko03010, 35 proteins at 1 mg/L C_60_), suggesting an increase in the production of newly synthesised organelle proteins which must find its way from site of production in the cytosol to the organelle where it functions. It was not feasible to quantify changes in cellular components in the digestive gland during this study; however, we can postulate from prior studies that observed changes may be linked to changes in the mitochondria. Mitochondria are essential eukaryotic organelles required for a range of metabolic, signalling and development processes. Using fullerenol, a polyhydroxylated fullerene derivative, Yang et al. demonstrated significant changes to isolated mitochondria via mitochondrial swelling, collapse of membrane potential, decreased of membrane fluidity and alterations to the ultrastructure [106]. The increase in protein production via the ribosome at the highest concentration may reflect the activation of a repair mechanism for damage to this structure. In a recent review, the main negative molecular and cellular responses associated with carbon nanotube (CNTs) in mammals were associated with oxidative stress which can promote inflammation, mitochondrial oxidation and activation of apoptosis [107]. Additionally, Zhang et al. reported on a loss in mitochondrial membrane potential in a mouse in vitro model, in association with increase in cellular ROS suggesting mitochondria associated apoptosis [105]. In a typical aquatic NP exposure, uptake is followed by localisation into the endosomes, lysosomes and digestive associated cells as well as the lumen of digestive tubules [22,27,108]. This NP exposure response can be followed by disruption or modification to mitochondrial activity [30]. Although the current study would support the hypothesis of mitochondrial damage/repair, further work will need to be carried out to verify.

#### 3.4.3. KEGG Pathway Enrichment

To further analyse the identified proteins per treatment, KEGG pathway analysis was performed. Using the bioconductor package clusterProfiler, protein sequences were assigned to DEPs (*p* < 0.05) and submitted to GhostKoala to obtained KEGG Orthology numbers (KO). In general, 52–56% of entries were successfully annotated with approximately 92% of annotations associated with the mollusca taxonomy. Variation between enrichment was described per treatment and concentration as follows:

**B[a]P**: at 5 µg/L exposure, 52 enriched processes were identified and include ribosome processes (26 genes), thermogenesis (19 genes), protein processing in endoplasmic reticulum (13 genes) and mTOR signalling pathway (nine genes). At 50 µg/L exposure, 38 pathways were enriched and ribosome (26 genes), protein processing in the endoplasmic reticulum (17 genes) and phagosome (13 genes). Finally, at 100 µg/L, 26 enriched processes were identified including ribosome (26 genes), RNA transport (16 genes), protein processing in the endoplasmic reticulum (16 genes), biosynthesis of amino acids (16 genes) and endocytosis (15 genes). The mTOR signalling pathway was not enriched at either 50 or 100 µg/L.

**Figure 7:**
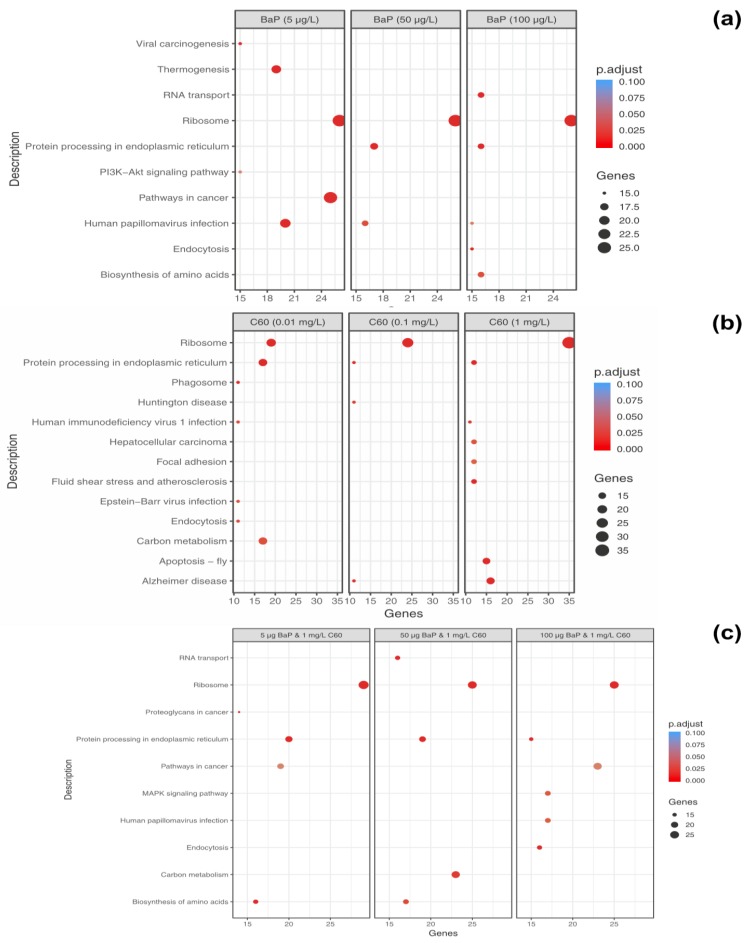
Dotplot of enriched KEGG pathways for differentially expressed genes (DEGs) (*p* < 0.05) that were common between concentrations of B[a]P (**a**), C_60_ (**b**) and a mixture of 5, 50 and 100 µg/L with 1 mg/L C_60_ (**c**). Along the *x*-axis, genes represent the number of genes identified as enriched in this particular pathway. The size and colour of each dot represents the gene number and adjustment *p* based on FDR correction.

The majority of enriched pathways identified can be grouped under genetic information processing, cellular processes, environmental information processing and metabolism. The top enriched pathways identified per concentration were plotted to identify commonalties and differences between differing concentrations of B[a]P (Figure 7a) based on genes identified in that pathway. Interestingly, unique pathways appear to be activated dependent on exposure concentration, with only the ribosome pathway consistently present and enriched at all concentrations potentially indicating the high degree of translation which may be occurring as a consequence of PAH exposure.

**Figure 8:**
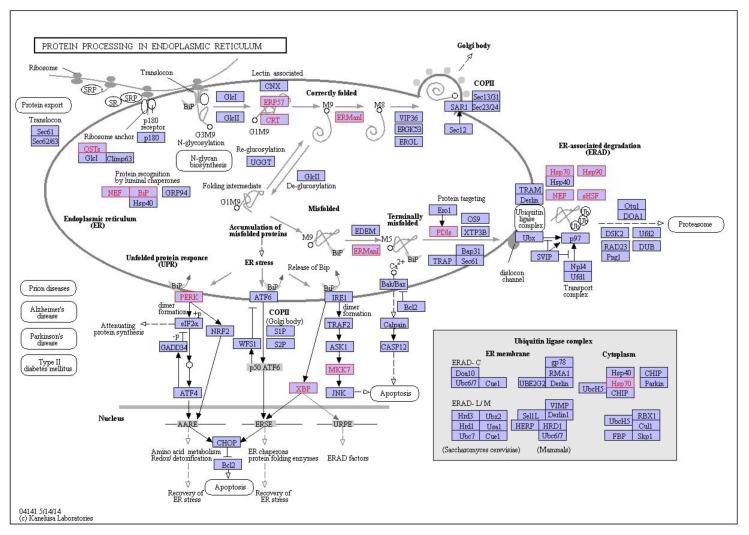
Interaction network of differentially expressed genes in the digestive gland of *M. galloprovincialis* involved in protein processing in the endoplasmic reticulum during exposure to 1 mg/L *n*C_60_. Genes which are differentially expressed during exposure are highlighted in red.

**C_60_**: at 0.01 mg/L exposure, 33 enriched pathways were identified while 12 enriched pathways were identified at 0.1 mg/L exposure and 35 enriched pathways identified at 1 mg/L exposure (*p* < 0.05, FDR = 5%). The top enriched pathways were illustrated in Figure 7b, with an absence of enrichment of certain pathways dependent on treatment concentration. For example, thermogenesis was only enriched at the highest concentration of 1 mg/L with 12 genes identified in the pathway. The ribosome is the top enriched pathway at all concentrations of C_60_ with 19 genes enriched at 0.01 mg/L exposure, 24 genes enriched at 0.1 mg/L exposure and 35 genes enriched at the highest concentration of 1 mg/L. This is closely followed by protein processing in endoplasmic reticulum, which is broadly comparable in terms of genes between 0.01 mg/L (17 genes), 0.1 mg/L (11 genes) and 1 mg/L (16 genes, Figure 8) exposure. The enriched pathways can be broadly grouped into predominantly genetic information processing, metabolism and cellular processes.

**Mixtures**: Under mixture scenario, C_60_ at a constant concentration of 1 mg/L was mixed with 5, 50 and 100 µg/L of B[a]P resulting in 50, 38 and 54 enriched pathways, respectively. At the lower mixture concentration of 5 µg/L B[a]P and C_60_, the top three enriched descriptive terms were related to the ribosome (29 genes), protein processing in endoplasmic reticulum (20 genes) and pathways in cancer (23 genes). At 50 µg/L B[a]P and C_60_, the top three enriched descriptive terms were related to the ribosome (23 genes), carbon metabolism (23 genes) and protein processing in endoplasmic reticulum (19 genes).

Finally, at 100 µg/L B[a]P and C_60_, the top three enriched descriptive terms were related to the ribosome (25 genes), pathways in cancer (23 genes) and mitogen-activated protein kinase signalling pathway (MAPK) signalling pathway (17 genes). Key genes consistently identified in the protein processing in the endoplasmic reticulum (irrespective of treatment) include *Hsp70*, *Hsp90*, *TRAP*, *PDIs* and *OSTs*. At the highest concentration of B[a]P and C_60_, genes identified in pathways in cancer include *GSTs*, *CASP3* and *Wnt*. The top pathways based on quantity of genes present in the pathway were presented in Figure 7 with clear trends towards an absence of enrichment in certain pathways based on mixture concentration, e.g., MAPK signalling, which is only present at the top exposure concentration combination.

KEGG pathway analysis can provide physiological pathway information for various experiments with prior studies using it to aid in identification of mode of action of environmental contaminants [77]. In the current study, irrespective of exposure conditions or concentrations, the top enriched pathway identified using KEGG was the Ribosome with 19–39 genes identified in the pathway dependent on treatment and concentration. This was followed by protein processing in the endoplasmic reticulum and carbon metabolism. The ribosome is a large complex molecule made of RNA and proteins that perform the essential task of protein synthesis in the cell. They also serve as the initiation point for several translation-associated functions including protein folding and degradation of defective or nonstop mRNAs. Previous studies have demonstrated a change in regulation of genes which encode ribosomal protein subunits following B[a]P exposure, with the suggestion that mRNA directed protein synthesis is reduced in mussels exposed to higher B[a]P loads [33]. Additionally, *M. galloprovincialis* has been shown to response to B[a]P exposure via changes in abundance of proteins related to synthesis and degradation, energy supply (via ATP) and structural proteins [77]. Proteomic results for B[a]P exposure to digestive gland tissue are in agreement with prior studies and support the observed trends identified using transcriptomic methodologies. In the second most enriched pathway (viz. protein processing in endoplasmic reticulum), three heat shock proteins viz. *HSP70, HSP90 and HSP40* and other molecular chaperones were identified dependent on exposure conditions. This is not surprising given that many Heat Shock Proteins (*HSPs)* function as molecular chaperones to protect damaged proteins from aggregation, unfold protein aggregates or refold damaged proteins or target them for efficient removal [109]. These proteins regulate cell response to oxidative stress with *HSP70* strongly upregulated by heat stress and toxic chemicals. *HSP70* plays several essential roles in cellular protein metabolism [110,111] while *HSP40* facilitates cellular recovery from adverse effects of damaged or misfolded proteins (proteotoxic stress). Changes in HSPs, in addition to up/down regulation of *HSP40, HSP70* and *HSP90* have typically been reported in response to thermal stress in bivalves [95,112,113] and other environmental contaminants such as B[a]P [33]. In general, the consistent enrichment of genes involved in the endoplasmic-reticulum associated protein degradation (ERAD) pathway suggest that aqueous fullerene exposure targets the cellular pathway involved in targeting misfolding proteins for ubiquitination (post-translational modification) and subsequent degradation by proteasomes (protein degrading complex, breaks peptide bonds). It is interesting to note the overlap between organismal response to fullerene exposure and that of organismal response to thermal stress. Observed enrichment pathways in the current study viz protein processing in endoplasmic reticulum, apoptosis, ubiquitin mediated proteolysis, endocytosis, spliceosome, and MAPK signalling pathway have been observed as differentially enriched in oysters as a response to thermal stress [112].

### 3.5. Notes

The lack of consensus regarding C_60_ toxicity may be partly due to limited studies which incorporate both a physiological and ecological approach. As a consequence, little is still known about NP bioavailability, mode of uptake, ingestion rates and actual internal concentrations related to ADME [27]. Generally, the greater the water solubility of fullerene aggregates (through e.g., stirring, surface modifications, sonication), the less the toxicity associated with the exposure [88]. Gomes et al. highlight that, while mussels represent a target for environmental exposure to nanoparticles, exposure duration may significantly contribute to NPs’ mediated toxicity [114]. As such, it is possible that the lack of differentially expressed proteins identified in this study is a factor of limited exposure duration. Limited exposure duration in the region of days or hours is common in the literature, and it would be of interest to explore long term exposure to NPs to look at the long-term impact and adaptation of mussels in the marine environment. Species specific responses to C_60_ are abundant in the literature and it would be remiss to not discuss how our results align with other marine invertebrates. Exposure to ROS can cause a range of reversible and irreversible modifications of protein amino acid side-chains which has been reviewed by Ghezzi and Bonetto [115]. Within the field of aquatic ecotoxicology, the toxic impact and potential mechanisms of single contaminant exposures have been extensively studied via laboratory experiments (in vivo, in vitro and in silico) and field monitoring. However, harder to predict is the effects of mixtures of pollutants in the environment. Biological damage observed cannot simply be linked to the actual environmental condition as mixtures of contaminants are known to exist in the aquatic ecosystem. This is further complicated with respect to nanomaterials due to their inherent properties which can amplify or negate the toxic effects of other compounds [75]. Complicated interactions may occur which make interpretation complex. For example, proteomic analysis of *Mytilus galloprovincialis* revealed that single Cu and B[a]P exposure in addition to a combination of the two generate different protein profiles with a non-additive profile [77]. Differences in mixture response compared to single exposure are likely to be related to individual chemical properties and toxicity mechanisms of B[a]P and C_60_, as has been noted in B[a]P co-exposed with various metals [116]. C_60_ concentration was kept constant with increasing concentrations of B[a]P in an experimental design that has been previously carried out using algae and crustacean species [22]. This may reflect limited proteome changes at the exposure concentrations, with concentrations of C_60_ in the range of 10–500 ppb have been reported to be 10 fold below the no observable adverse effect level (NOAEL) [117,118]. At 1 mg/L, an increase in Glutathione S-Transferase (GST) activity in the digestive gland has been reported [108]. C_60_ is known to bind to minor grooves of double stranded DNA and trigger unwinding and disruption of the DNA helix [100]. C_60_ adsorbs onto cell-membrane P-glycoprotein through hydrophobic interactions, but the stability and secondary structure of the protein are barely affected [119]. P-glycoprotein is present in *Mytilus galloprovincialis* [120]. C_60_ and its derivatives are known to impact DNA and RNA in terms of stability, replication and reactivity in addition to structural stabilisation [121,122]. In a recent study, Canesi et al. determined that C_60_ fullerene exposure to *Mytilus galloprovincialis* hemocytes did not induce significant cytoxicity, and instead stimulated immune and inflammatory parameters such as lysozyme release, oxidative burst and nitric oxide (NO) production [10]. Nanomaterial suspensions can induce inflammatory processes in bivalve hemocytes akin to those observed in vertebrate cells [10]. Results from mammalian studies suggest that C_60_ fullerene exposure results predominantly in inflammatory responses [123].

## 4. Conclusions

This study has confirmed our hypothesis of an interaction between B[a]P and C_60_, two ubiquitous environmental contaminants. We demonstrated for the first time an apparent antagonistic relationship at the genotoxic and the proteome expression level, which is not visible at lower exposure concentrations. This response is not explained by expected strong sorption of B[a]P on C_60_ as no difference in bioaccumulation was noted, but rather by the free radical scavenger propriety of C_60_. No Trojan horse effects were observed for uptake or toxicity of the co-contaminants B[a]P in interaction with C_60_. Proteome profile is dependent on concentration and treatment. The exposure to the three conditions had overlap and common mechanisms of response irrespective of differences in mode of action. The provided list of condition specific differentially expressed proteins and enriched pathways (Spreadsheet S1) may represent a step towards definitively identifying mode of action of these compounds in bivalves when combined with other OMICs based approaches. It should be noted that the antagonistic proteome response observed in the current study between B[a]P and C_60_ is based on a single concentration of the fullerene and as such represents a general overview of toxicological behaviour. It is possible that that this antagonistic interaction will change when another dose range is selected [90]. Gomes et al. previously highlighted that, while mussels represent a target for environmental exposure to nanoparticles, exposure duration may significantly contribute to NPs’ mediated toxicity [114]. As such, further work must be carried out to explore mixture effects at different concentrations and over differing exposure duration.

## Figures and Tables

**Table 1:** Analysis of combined effects of B[a]P and C_60_ on DNA damage based on Interaction Factors (IF).

Treatments	IF for DNA Damage (Comet Assay)
BaP 5 µg/L + C_60_ 1 mg/L	−7.48 ± 6.63
BaP 50 µg/L + C_60_ 1 mg/L	−10.39 ± 3.50
BaP 100 µg/L + C_60_ 1 mg/L	−12.69 ± 6.05 *

Interaction Factor ± 95% Confidence Limit /√2. * indicates significance at the 5% level. A negative IF indicates antagonism; an IF of 0 indicates additivity; and a positive IF indicates synergism. Statistical significance was determined by testing for overlap between the mixture IF ± 95% CL/√2 and the predicted additive value for C_60_ and B[a]P, assumed to have an IF = 0 ± 95% CL/√2, where the confidence limit is derived from the SEM_(add)_ value for the additive C_60_ and B[a]P.

**Table 2:** Significantly expressed proteins of B[a]P, C_60_ and mixture (5–100 µg/L and 1 mg/L C_60_). Species id’s are as follows: 6573 = *Mizuhopecten yessoensis*, 6551 = *Mytilus trossulus*, 29159 = *Crassostrea gigas* and 94323 = *Crassostrea ariakensis*.

Treatment	Species	Protein Name	UNIPROTKB	GO Annotation	Regulation
B[a]P (5 µg/L)	6573	Arrestin domain-containing protein 3	A0A210PE39		Up
B[a]P (5 µg/L)	6573	Orexin receptor type 2	A0A210PSC6	GO:0004930, GO:0016021	Up
B[a]P (5 µg/L)	6573	Ran-specific GTPase-activating protein	A0A210Q6H5	GO:0005622, GO:0046907	Up
B[a]P (50 µg/L)	6573	5-hydroxytryptamine receptor 1A-alpha	A0A210R4M3	GO:0004993, GO:0005887, GO:0008283, GO:0042310, GO:0046883, GO:0050795	Down
B[a]P (50 µg/L)	6573	Adenylate kinase isoenzyme 5	A0A210QMB2	GO:0005524, GO:0006139, GO:0019205	Up
B[a]P (50 µg/L)	6573	Uncharacterised protein	A0A210Q912		Up
B[a]P (100 µg/L)	6573	Helicase with zinc finger domain 2	A0A210PQ46	GO:0004386, GO:0030374	Up
B[a]P (100 µg/L)	29159	Peroxiredoxin-4	K1QLH0	GO:0005623, GO:0045454, GO:0051920	Up
B[a]P (100 µg/L)	29159	Hypoxia up-regulated protein 1	K1QBF7	GO:0005524	Up
B[a]P (5 µg/L) + C60 (1 mg/L)	94323	Ras-like GTP-binding protein RHO	H9LJA2	GO:0003924, GO:0005525, GO:0005622, GO:0007264	Up
B[a]P (5 µg/L) + C60 (1 mg/L)	29159	Zinc finger CCCH domain-containing protein 13	K1PKC9	GO:0046872	Up
B[a]P (5 µg/L) + C60 (1 mg/L)	29159	Myosin heavy chain, non-muscle (Fragment)	K1QXX7	GO:0003774 GO:0003779, GO:0005524 GO:0016459	Up
B[a]P (50 µg/L) + C60 (1 mg/L)	6551	Ribosomal protein S20	A0A077H0N2	GO:0003723, GO:0003735, GO:0006412, GO:0015935	Down
B[a]P (50 µg/L) + C60 (1 mg/L)	6573	Nucleolar and coiled-body phosphoprotein 1	A0A210Q9W0	GO:0005730	Down
B[a]P (50 µg/L) + C60 (1 mg/L)	29159	Tripartite motif-containing protein 2	K1QBD4	GO:0005622, GO:0008270	Up
B[a]P (100 µg/L) + C60 (1 mg/L)	6573	Ran-specific GTPase-activating protein	A0A210Q6H5	GO:0005622, GO:0046907	Down
B[a]P (100 µg/L) + C60 (1 mg/L)	29159	Uncharacterized protein	K1R543		Down

**Table 3:** Subset of enriched Gene Ontology (GO) terms with an family wise error (FWER) threshold of 1% (or 0.01) following B[a]P (5–100 µg/L ), C_60_ (0.01–1 mg/L) and a mixture of B[a]P (5–100 µg/L) and C_60_ (1 mg/L) treatments. Cellular component and biological processes are abbreviated to CC and BP, respectively.

Treatment	Ontology	GO-ID	GO-ID Name	FWER
B[a]P (100 µg/L)	BP	GO:0006139	Nucleobase-containing compound metabolic process	0.01
B[a]P (100 µg/L)	BP	GO:0006725	Cellular aromatic compound metabolic process	0.01
B[a]P (100 µg/L)	BP	GO:0034641	Cellular nitrogen compound metabolic process	0.01
B[a]P (100 µg/L)	BP	GO:0046483	Heterocycle metabolic process	0.01
B[a]P (100 µg/L)	BP	GO:0090304	Nucleic acid metabolic process	0.01
B[a]P (100 µg/L)	BP	GO:1901360	Organic cyclic compound metabolic process	0.01
C_60_ (0.01 mg/L)	BP	GO:0000226	Microtubule cytoskeleton organization	0.01
C_60_ (0.1 mg/L)	CC	GO:0031974	Membrane-enclosed lumen	0.01
C_60_ (0.1 mg/L)	CC	GO:0043233	Organelle lumen	0.01
C_60_ (0.1 mg/L)	CC	GO:0070013	Intracellular organelle lumen	0.01

## Supplementary Materials

The following are available online at https://www.mdpi.com/2079-4991/9/7/987/s1, Figure S1: (a) bright-field TEM and (b) intensity-weighted particle size distribution of nC60 aggregates present in mussel-exposed seawater, Figure S2: (a) bright-field TEM and (b) point EDX spectroscopy analysis of Er_3_N@C_80_, Figure S3: Dark-field STEM and EDX spectroscopy mapping analysis of Er_3_N@C_80_, confirming the necessity for spectroscopy to confirm the presence of labelled fullerenes, using the characteristic X-rays emitted from Er upon electron irradiation, Figure S4: (a,c,e) dark-field STEM and (b,d,f) corresponding point EDX spectroscopy analysis of cross-sections of mussel digestive gland exposed to Er_3_N@C_80_, Figure S5: Volcano plots representing the differentially expressed proteins with exposure to B[a]P, C_60_ or a mixture of the two (5–50–100 µg/L B[a]P, 1 mg/L C_60_), Table S1: The influence of benzo[a]pyrene (B[a]P) of the hydrodynamic diameter (d_H_) of *n*C_60_ in mussel-exposed seawater as determined by DLS, Table S2: The concentration of B[a]P in seawater at T0, day 1 and day 3, Table S3: The concentration of nC_60_ in seawater at T0, day 1 and day 3, Spreadsheet S1: Full list of DEPs, enriched Gene Ontology (GO) terms and KEGG pathways, R script S1: R script used for proteomics analysis.
