# Supplementary material for: Antagonistic Interactions between Benzo[a]pyrene and Fullerene (C60) in Toxicological Response of Marine Mussels
Source: Nanomaterials (Basel). 2019 Jul 8;9(7):987. doi: 10.3390/nano9070987 (PMC6669530; doi:10.3390/nano9070987)
Supplement: Supplementary file 1 [file nanomaterials-09-00987-s001.zip › nanomaterials-513790/R_Script_S1.pdf]

Proteomics Analysis: Trojan Horse Project

##Load Packages

```
library(dplyr)
library(MSGFplus)
library(MSnbase)
library(MSnID)
library(vsn)
library(imputeLCMD)
library(msmsEDA)
library(msmsTests)
library(clusterProfiler)
library(GOfuncR)
```

#Combine contaminants and protein file Create common contaminants and protein fasta. Contaminants file is downloaded from ftp://ftp.thegpm.org/fasta/cRAP (publicly availble list of contaminants maintained by lab group at https://www.thegpm.org/cRAP/) and placed in folder called fasta for convenience with UNIPROT bivavlia fasta.

```
library(seqRFLP)
file.cat(dir = "fasta", appendix = ".fasta", file = "combined_filter_file.fasta")
```

#Set processing parameters

```
tro.par = msgfPar(database = "combined_filter_file.fasta", tolerance = "10 ppm",
    tda = TRUE, instrument = "HighRes", enzyme = "Trypsin", ntt = 2,
    protocol = 0)

nMod(tro.par) = 2
mods(tro.par)[[1]] <- msgfParModification("Carbamidomethyl",
    composition = "C2H3N1O1", residues = "C", type = "fix", position = "any")

mods(tro.par)[[2]] <- msgfParModification(name = "Oxidation",
    mass = 15.994915, residues = "M", type = "opt", position = "any")

# set directory where files are located
files.b = list.files(pattern = ".mzML", full.names = TRUE, recursive = TRUE)
# Run identification
pre_process = runMSGF(tro.par, files.b, import = FALSE)
```

##Start project and import data

```
t1 = c("20171124VS37.mzML")
q1 = c("20171124VS37.mzid")
bap_0a = readMSData(t1, mode = "onDisk")
bap_0a = addIdentificationData(bap_0a, q1)

t2 = c("20171124VS38.mzML")
q2 = c("20171124VS38.mzid")
bap_0b = readMSData(t2, mode = "onDisk")
bap_0b = addIdentificationData(bap_0b, q2)

t3 = c("20171124VS39.mzML")
q3 = c("20171124VS39.mzid")
```

```
bap_0c = readMSData(t3, mode = "onDisk")
bap_0c = addIdentificationData(bap_0c, q3)

t4 = c("20171124VS40.mzML")
q4 = c("20171124VS40.mzid")
bap_5a = readMSData(t4, mode = "onDisk")
bap_5a = addIdentificationData(bap_5a, q4)

t5 = c("20171124VS41.mzML")
q5 = c("20171124VS41.mzid")
bap_5b = readMSData(t5, mode = "onDisk")
bap_5b = addIdentificationData(bap_5b, q5)

t6 = c("20171124VS42.mzML")
q6 = c("20171124VS42.mzid")
bap_5c = readMSData(t6, mode = "onDisk")
bap_5c = addIdentificationData(bap_5c, q6)

t7 = c("20171124VS43.mzML")
q7 = c("20171124VS43.mzid")
bap_50a = readMSData(t7, mode = "onDisk")
bap_50a = addIdentificationData(bap_50a, q7)

t8 = c("20171124VS44.mzML")
q8 = c("20171124VS44.mzid")
bap_50b = readMSData(t8, mode = "onDisk")
bap_50b = addIdentificationData(bap_50b, q8)

t9 = c("20171124VS45.mzML")
q9 = c("20171124VS45.mzid")
bap_50c = readMSData(t9, mode = "onDisk")
bap_50c = addIdentificationData(bap_50c, q9)

t10 = c("20171124VS46.mzML")
q10 = c("20171124VS46.mzid")
bap_100a = readMSData(t10, mode = "onDisk")
bap_100a = addIdentificationData(bap_100a, q10)

t11 = c("20171124VS47.mzML")
q11 = c("20171124VS47.mzid")
bap_100b = readMSData(t11, mode = "onDisk")
bap_100b = addIdentificationData(bap_100b, q11)

t12 = c("20171124VS48.mzML")
q12 = c("20171124VS48.mzid")
bap_100c = readMSData(t12, mode = "onDisk")
bap_100c = addIdentificationData(bap_100c, q12)
```

##Batch process samples

```
# Batch processing:
nms = c(paste0("bap_", 0, c("a", "b", "c")), paste0("bap_", 5,
    c("a", "b", "c")), paste0("bap_", 50, c("a", "b", "c")),
    paste0("bap_", 100, c("a", "b", "c")))
```

```
tmp <- sapply(nms, function(.bap) {
    cat("Processing", .bap, "... ")
    x <- get(.bap, envir = .GlobalEnv)
    x = quantify(x, method = "SI")
    x <- topN(x, groupBy = fData(x)$DatabaseAccess, n = 3)
    nPeps <- nQuants(x, fData(x)$DatabaseAccess)
    x <- combineFeatures(x, fData(x)$DatabaseAccess, redundancy.handler = "unique",
        fun = "sum", na.rm = TRUE, CV = FALSE)
    exprs(x) <- exprs(x) * (3/nPeps)
    x <- updateFvarLabels(x, .bap)
    varnm <- sub("bap", "bap", .bap)
    assign(varnm, x, envir = .GlobalEnv)
    cat("done\n")
})
```

##Combining biological/technical replicates, filtering and normalisation

```
ctrl = combine(bap_0a, bap_0b)
ctrl = combine(ctrl, bap_0c)
ctrl = normalise(ctrl, "vsn")
ctrl = filterNA(ctrl, pNA = 1/3)
ctrl = impute(ctrl, method = "knn")
sampleNames(ctrl) = c("CTRL.a", "CTRL.b", "CTRL.c")

bap5 = combine(bap_5a, bap_5b)
bap5 = combine(bap5, bap_5c)
bap5 = normalise(bap5, "vsn")
bap5 = filterNA(bap5, pNA = 1/3)
bap5 = impute(bap5, method = "knn")
sampleNames(bap5) = c("BaP.5a", "BaP.5b", "BaP.5c")

bap50 = combine(bap_50a, bap_50b)
bap50 = combine(bap50, bap_50c)
dim(bap50)
bap50 = normalise(bap50, "vsn")
bap50 = filterNA(bap50, pNA = 1/3)
bap50 = impute(bap50, method = "knn")
sampleNames(bap50) = c("BaP.50a", "BaP.50b", "BaP.50c")

# Biological combination for 100 ug BaP exposure
bap100 = combine(bap_100a, bap_100b)
bap100 = combine(bap100, bap_100c)
bap100 = normalise(bap100, "vsn")
bap100 = filterNA(bap100, pNA = 1/3)
dim(bap100)
bap100 = impute(bap100, method = "knn")
sampleNames(bap100) = c("BaP.100a", "BaP.100b", "BaP.100c")
```

#Differential expression For differential analysis, quasi likelihood based GLM regression (msms.glm.qlll) was used to discover DEP between 2 conditions. This is a distribution free model which allows for overdispersion and should be used where an appreciable source of biological variability is expected.

```
# DEP analysis
combo1 = combine(ctrl, bap5)
dim(combo1)
```

```r
combo1 = impute(combo1, "QRILC")

## COMBO1 comparison
pData(combo1)$Group = factor(rep(c("0", "5"), each = 3))
pData(combo1)$Treat = c(rep("untreated", 3), rep("treated", 3))

combo1a = pp.msms.data(combo1)
null.f = "y~1"
alt.f = "y~Treat"
norm.count = exprs(combo1a)
disp2 = apply(norm.count, 2, sum)

ql.1 = msms.glm.qlll(combo1a, alt.f, null.f, div = disp2)
head(ql.1)
sum(ql.1$p.value < 0.05)
ql.1$adjp = p.adjust(ql.1$p.value, method = "BH")
sum(ql.1$adjp <= 0.01)
```

##Volcano Plots

```r
with(ql.1, plot(LogFC, -log10(p.value), pch = 20, main = "5 ug BaP",
    xlim = c(-1, 1), font.lab = 2))
with(subset(ql.1, adjp < 0.05), points(LogFC, -log10(p.value),
    pch = 20, col = "red"))
with(subset(ql.1, adjp < 0.01), points(LogFC, -log10(p.value),
    pch = 20, col = "blue"))
abline(h = 1.65, col = "red", lwd = 3, lty = 2)
text(x = 0.95, y = 1.85, labels = "P.adj < 0.05", font = 2, col = "red")
abline(h = 3.7, col = "blue", lwd = 3, lty = 2)
text(x = 0.95, y = 3.9, labels = "P.adj < 0.01", font = 2, col = "blue")
```

#KEGG analysis

```r
bap5 <- read.csv("G:/Trojan_horse_proteomics_data/Analysis/Bap/DEP/ctrl_5bap.csv")
head(bap5)
bap5b = bap5[, 7:8]  #select column with kegg number and p.value
bap5c = subset(bap5b, p.value < 0.05)  #subset to signfigant proteins
bap5d = bap5c[, 1]  #subset to signfigant kegg values

kk5 = enrichKEGG(gene = bap5d, organism = "ko", ueCutoff = 0.05,
    pAdjustMethod = "BH")
```

#Gene ontology anaalysis

```r
# subset dep data set
head(ctrl_5bap)
geneList5 = ctrl_5bap[, 4:5]  #selecting gene symbol and log change columns
colnames(geneList5) = c("gene", "score")  #select gene symbol and logfc
head(geneList5)

geneList5b = geneList5 %>% distinct(gene, .keep_all = TRUE)  #remove duplicates
geneList5b$gene = as.character(geneList5b$gene)  #data must be character
head(geneList5b)

# load custom annotations as described above.
custom = read.csv("custom_mollusc_annotations.csv")  #download GO data from UNIPROTKB
```

```r
custom$gene = as.character(custom$gene)
custom$go_id = as.character(custom$go_id)

res = go_enrich(geneList5b, test = "wilcoxon", annotations = custom)
# GO enrichment from analysis (ordered by FWER for
# overrepresentation of candidate genes)
head(res[[1]])
# top GOs from every GO-domain
by(res[[1]], res[[1]][, "ontology"], head)
# top GOs per domain (top 5)
st1 = res[[1]]
by(st1, st1$ontology, head, n = 5)

View(res$results)
# with this, FWER is more stringent then FDR and so i select
# candidates less then 0.05 as signifigant.
```
