# Supplementary material for: Antagonistic Interactions between Benzo[a]pyrene and Fullerene (C60) in Toxicological Response of Marine Mussels
Source: Nanomaterials (Basel). 2019 Jul 8;9(7):987. doi: 10.3390/nano9070987 (PMC6669530; doi:10.3390/nano9070987)
Supplement: Supplementary file 1 [file nanomaterials-09-00987-s001.zip › nanomaterials-513790/Supplementary-materials.pdf]

Supplementary materials

Antagonistic Interactions between Benzo[a]pyrene and Fullerene (C_{60}) in Toxicological Response of Marine Mussels

Audrey Barranger [1,†,‡], Laura M. Langan [1,†,#], Vikram Sharma [2], Graham A. Rance [3,4], Yann Aminot [5], Nicola J. Weston [4], Farida Akcha [6], Michael N. Moore [1,7,8], Volker M. Arlt [9,10], Andrei N. Khlobystov [3,4], James W. Readman [5] and Awadhesh N. Jha [1,*]

[1] School of Biological and Marine Sciences, University of Plymouth, PL4 8AA Plymouth, UK
[2] School of Biomedical Sciences, University of Plymouth, PL4 8AA Plymouth, UK
[3] School of Chemistry, University of Nottingham, University Park, NG7 2RD Nottingham, UK
[4] Nanoscale and Microscale Research Centre, University of Nottingham, University Park, NG7 2RD Nottingham, UK
[5] Centre for Chemical Sciences, University of Plymouth, PL4 8AA Plymouth, UK
[6] Ifremer, Laboratory of Ecotoxicology, F-44311, Nantes Cedex 03, France
[7] Plymouth Marine Laboratory, Prospect Place, The Hoe, PL1 3HD Plymouth, UK
[8] European Centre for Environment & Human Health (ECEHH), University of Exeter Medical School, Knowledge Spa, Royal Cornwall Hospital, TR1 3LJ Cornwall, UK
[9] Department of Analytical, Environmental and Forensic Sciences, King's College London, MRC-PHE Centre for Environmental & Health, SE1 9NH London, UK
[10] NIHR Health Protection Research Unit in Health Impact of Environmental Hazards at King's College London in partnership with Public Health England and Imperial College London, SE1 9NH London, UK
* Correspondence: a.jha@plymouth.ac.uk; Tel.: +44-1752-584-633
‡ Present Address: Université de Rennes 1/Centre National de la Recherche Scientifique, UMR 6553 ECOBIO, Rennes, F-35000, France
# Present Address: Department of Environmental Science, Baylor University, 76706 Waco, TX, USA
† These authors contributed equally to this work.

S1. Characterisation of Fullerenes in Seawater

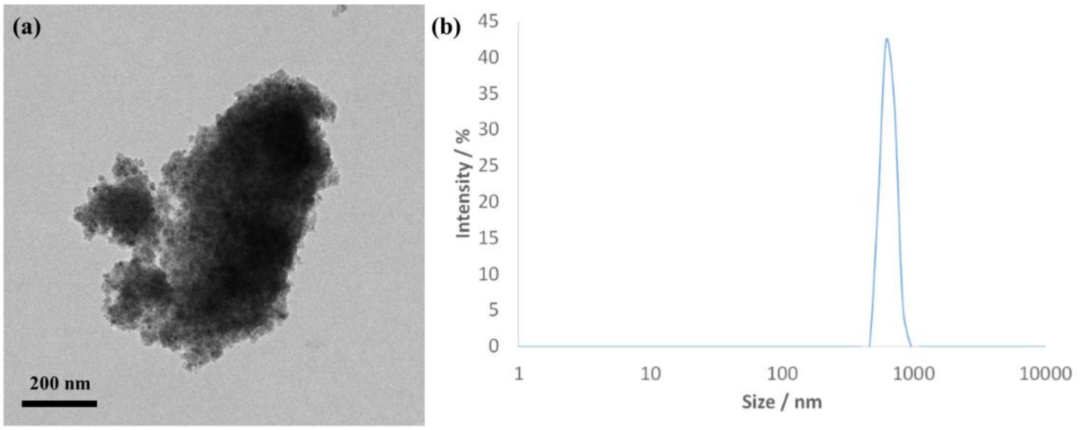

Figure S1. (a) Bright-field TEM and (b) intensity-weighted particle size distribution of *n*C_{60} aggregates present in mussel-exposed seawater. The electron micrograph (a) shows a typical *n*C_{60} aggregate ~600 nm in diameter, consistent with the hydrodynamic diameter (653 ± 87 nm) obtained from light scattering analysis (b). The maximum possible concentration of *n*C_{60} in seawater is 0.1 mg mL^{-1}, which is significantly higher than that utilised in exposure experiments, but within the range detectable by the instrument. Control light scattering measurements of seawater in the absence of *n*C_{60} yielded no measurable scatterers.

Table S1. The influence of benzo(a)pyrene (B[a]P) of the hydrodynamic diameter (d_H) of *n*C_{60} in mussel-exposed seawater as determined by DLS. The addition of B[a]P results in a subtle decrease in the mean size of aggregates; however, the decrease is small and likely insignificant given the broad range of sizes observed within the respective size distributions.

B[a]P (μg/L)	d_H (nm)
0	653 ± 87
5	601 ± 62
50	555 ± 64
100	543 ± 55

S2. Concentration of B[a]P and C_{60} in Seawater

Table S2. The concentration of B[a]P in seawater at T0, day 1 and day 3. Data are means SE (*n* = 3).

Treatments	B[a]P concentration (μg L^{-1})		
	T0	Day 1	Day 3
Solvent control	< 0.25	< 0.25	< 0.25
B[a]P 5 μg L^{-1}	4.8 ± 1.8	1.3 ± 2.0	0.1 ± 0.1
B[a]P 50 μg L^{-1}	52.9 ± 7.3	7.3 ± 4.1	0.7 ± 0.4
B[a]P 100 μg L^{-1}	94.4 ± 23.4	6.1 ± 4.9	1.5 ± 1.2
C_{60} + B[a]P 5 μg L^{-1}	6.2 ± 1.2	0.9 ± 0.9	0.1 ± 0.1
C_{60} + B[a]P 50 μg L^{-1}	52.0 ± 15.8	5.1 ± 6.7	1.3 ± 1.5
C_{60} + B[a]P 100 μg L^{-1}	102.0 ± 21.5	15.4 ± 16.0	2.5 ± 1.6

Table S3. The concentration of nC_{60} in seawater at T0, day 1 and day 3. Data are means SE (n = 3).

**Treatments**	**C_{60} concentration (µg L^{-1})**		
	**T0**	**Day 1**	**Day 3**
Solvent control	< 0.2	< 0.2	< 0.2
C_{60} 0.01 mg L^{-1}	7.3 ± 1.8	< 0.2	< 0.2
C_{60} 0.1 mg L^{-1}	63.8 ± 11.9	0.4 ± 0.6	< 0.2
C_{60} 1 mg L^{-1}	427.6 ± 45.3	0.5 ± 0.5	< 0.2
C_{60} + B[a]P 5 µg L^{-1}	518.3 ± 48.6	0.9 ± 0.6	0.5 ± 0.7
C_{60} + B[a]P 50 µg L^{-1}	477.5 ± 107.9	2.5 ± 4.3	< 0.2
C_{60} + B[a]P 100 µg L^{-1}	604.1 ± 52.0	2.7 ± 3.9	< 0.2

S3. Transmission Electron Microscopy Analysis of Labelled Fullerenes

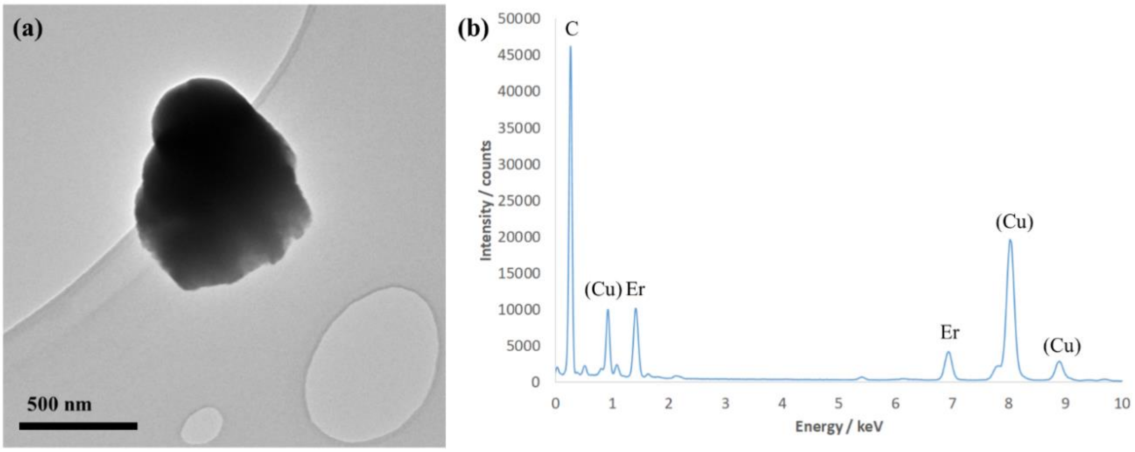

Figure S2. (a) Bright-field TEM and (b) point EDX spectroscopy analysis of $Er_3N@C_{80}$. The dark features (which comprise high atomic number elements) in the bright-field TEM images are used to visually locate nanoscale species of interest. This analysis confirms the presence of Er from the characteristic M and Lα lines at 6.95 and 1.41 eV, respectively. The presence of Cu, plus trace quantities of Cr and Au, in the EDX spectrum is associated with the TEM grid and column assembly. The sample was prepared by drop casting from methanolic suspension.

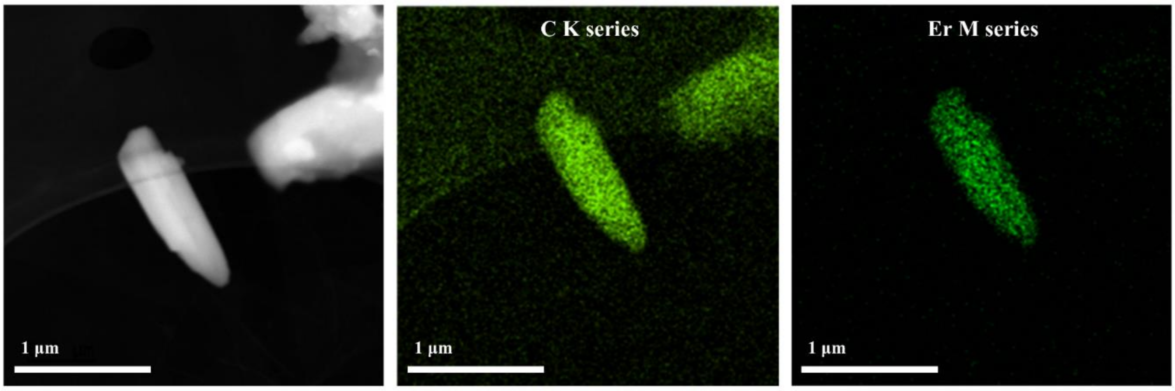

Figure S3. Dark-field STEM and EDX spectroscopy mapping analysis of $Er_3N@C_{80}$, confirming the necessity for spectroscopy to confirm the presence of labelled fullerenes, using the characteristic X-rays emitted from Er upon electron irradiation. The sample was prepared by drop casting from methanolic suspension.

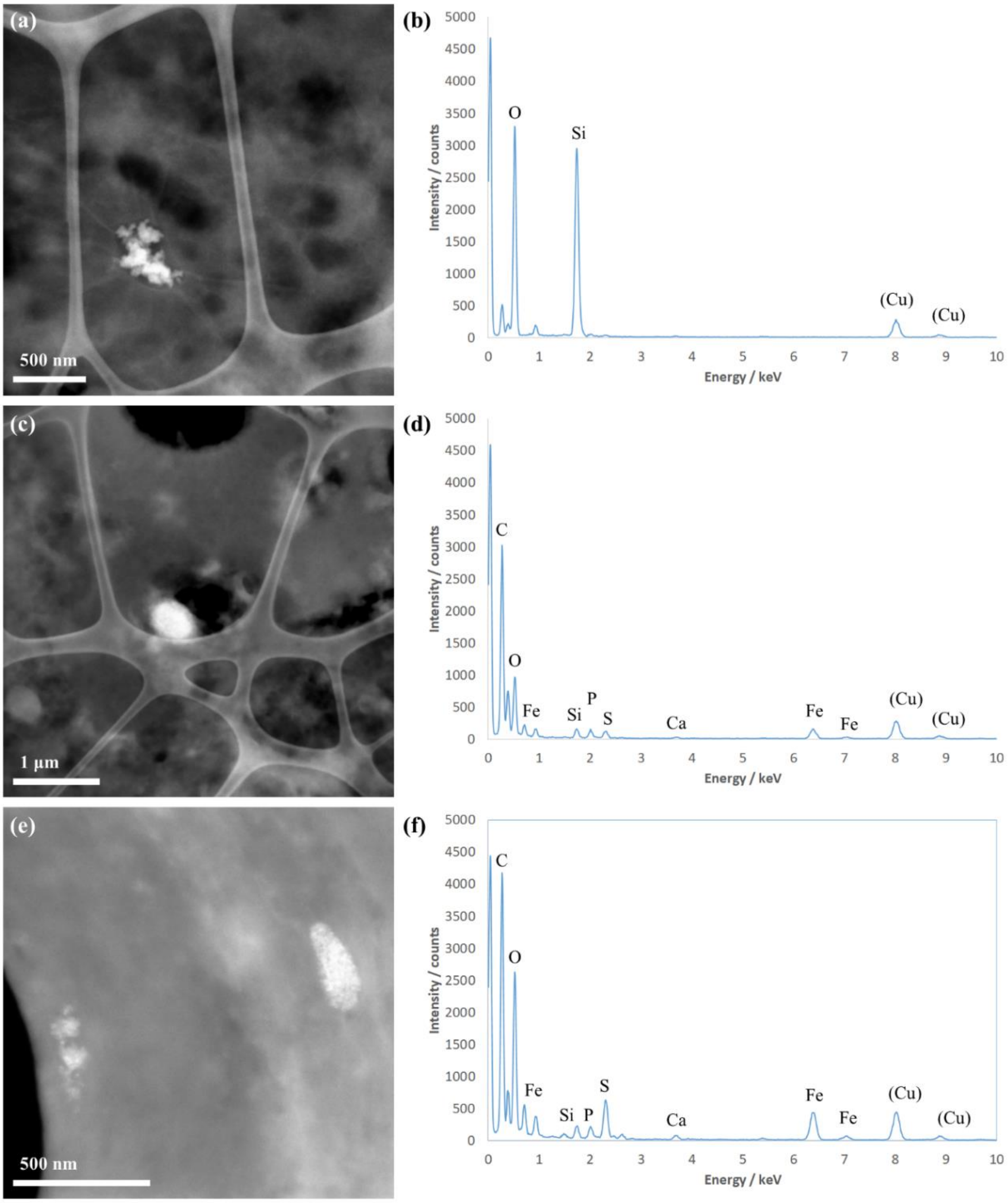

Figure S4. (a,c,e) Dark-field STEM and (b,d,f) corresponding point EDX spectroscopy analysis of cross-sections of mussel digestive gland exposed to $Er_3N@C_{80}$. The bright features (which comprise high atomic number elements) in the dark-field STEM images are used to visually locate nanoscale species of interest. The EDX spectra collected from these explicit locations confirm the presence of silica (b) and iron (d and f) as typical environmental contaminants, but no evidence for the uptake of labelled fullerene aggregates was directly observed. However, the presence of labelled fullerenes within the mussel digestive gland was confirmed by whole tissue ICP-MS analysis and therefore the absence of evidence from our STEM-EDX investigations implies that the labelled fullerenes are dispersed at potentially the near single molecule level, below the level of identification by imaging or sensitivity of *in situ* spectroscopy. The presence of Cu in the EDX spectra is associated with the specimen grid and instrument column assembly.

S4. Proteomics

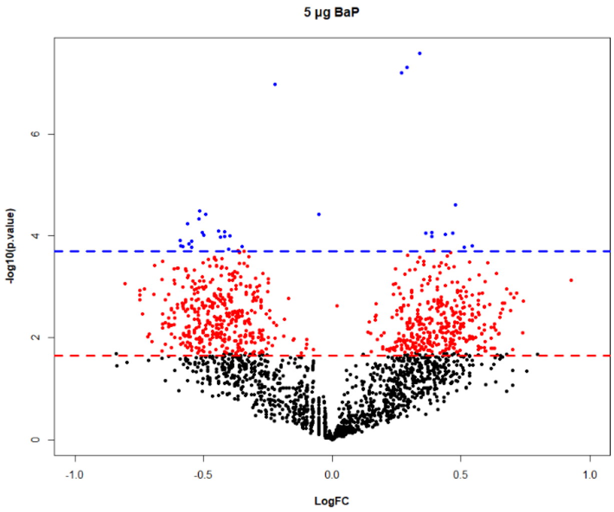

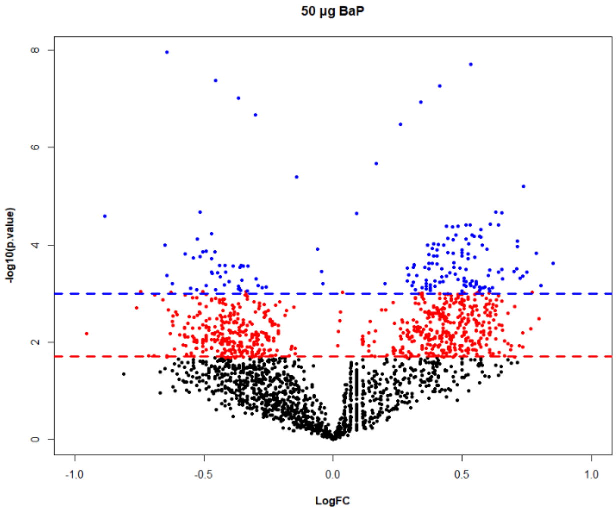

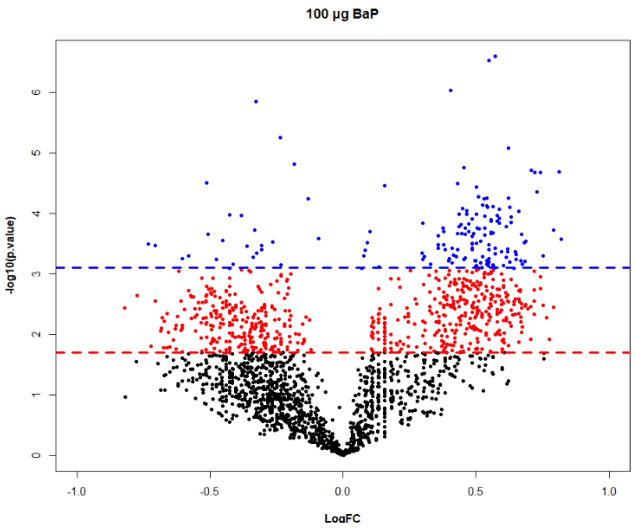
100 µg BaP
-log10(p.value)
0
1
2
3
4
5
6
-1.0
-0.5
0.0
0.5
1.0
LogFC

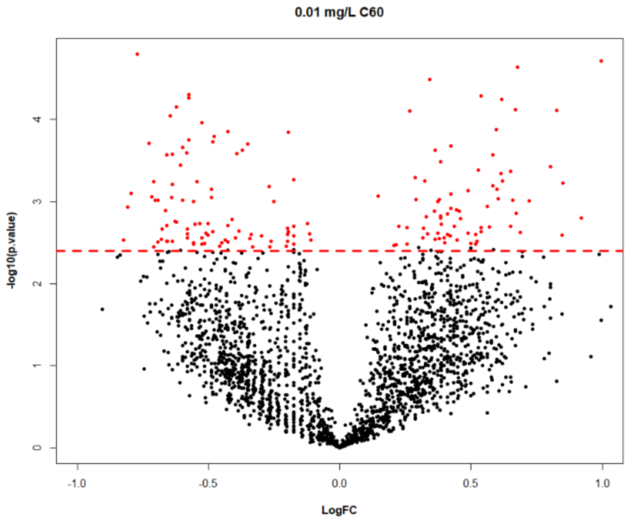
0.01 mg/L C60
-log10(p.value)
0
1
2
3
4
-1.0
-0.5
0.0
0.5
1.0
LogFC

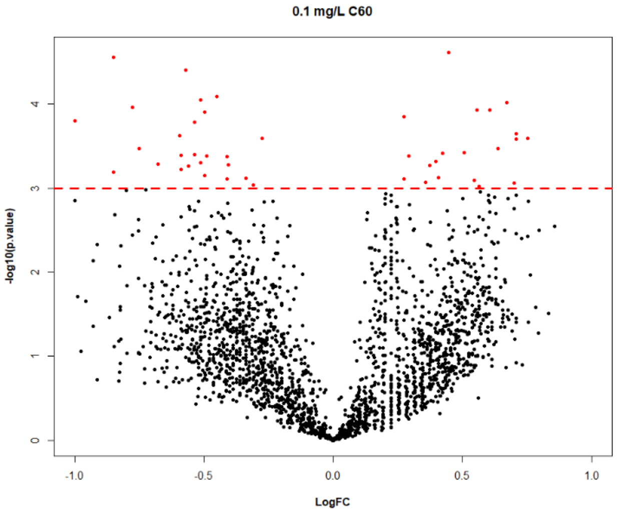
0.1 mg/L C60
-log10(p.value)
0
1
2
3
4
-1.0
-0.5
0.0
0.5
1.0
LogFC

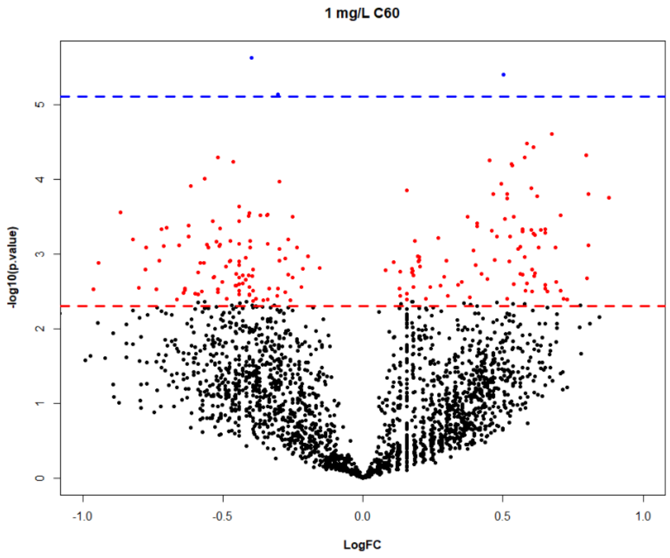
1 mg/L C60
-log10(p.value)
LogFC
-1.0
-0.5
0.0
0.5
1.0
0
1
2
3
4
5

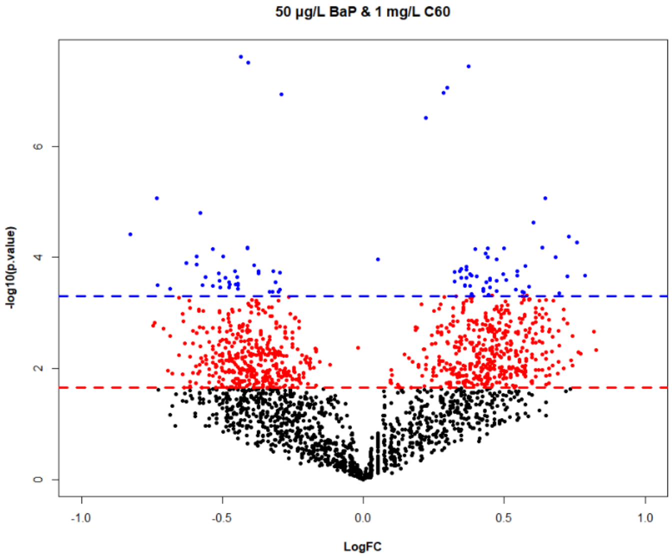
50 µg/L BaP & 1 mg/L C60
-log10(p.value)
LogFC
-1.0
-0.5
0.0
0.5
1.0
0
2
4
6

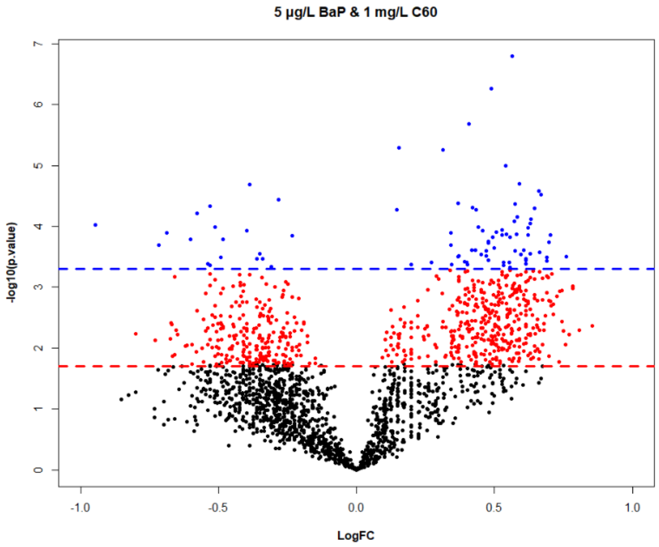
5 µg/L BaP & 1 mg/L C60
-log10(p.value)
LogFC
-1.0
-0.5
0.0
0.5
1.0
0
1
2
3
4
5
6
7

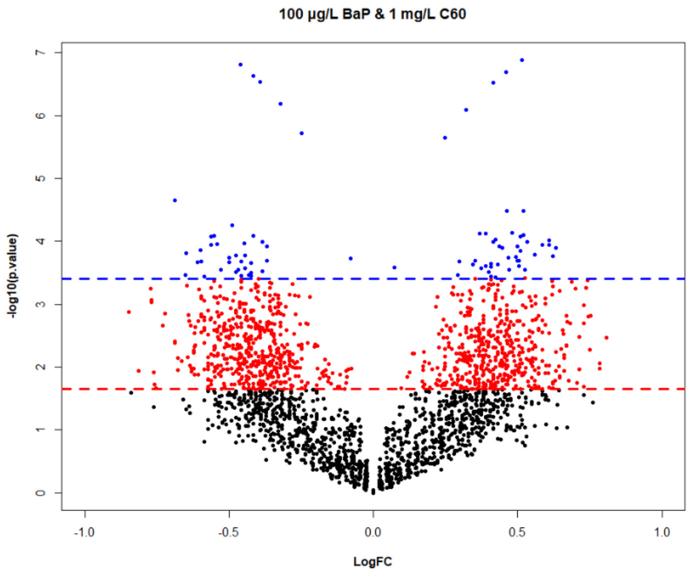

Figure S5: Volcano plots representing the differentially expressed proteins with exposure to B[a]P, C_{60} or a mixture of the two (5–50–100 µg/L B[a]P 1 mg/L C_{60}). Red dots represent DEPs ($p < 0.05$) with an FDR of 5% while blue dots represent DEPs with an FDR of 1%. Red and blue lines indicating the location of the dots have been added for clarity. Black dots represent unique proteins which are not differentially expressed.
